# Toward New Therapeutics for Visceral Leishmaniasis: Efficacy and Mechanism of Action of Amides Inspired by Gibbilimbol B

**DOI:** 10.1021/acsomega.4c05510

**Published:** 2024-10-08

**Authors:** Fabio Navarro Baltazar, Maiara Amaral, Maiara Maria Romanelli, Erica Valadares de Castro Levatti, Fernanda Fonseca Ramos, Luiz Paulo Melchior de Oliveira Leão, Daniela Aparecida Chagas-Paula, Marisi Gomes Soares, Danielle Ferreira Dias, Cecilia M. S. Q. Aranha, João Paulo dos Santos Fernandes, Joao Henrique Ghilardi Lago, Andre Gustavo Tempone

**Affiliations:** † Pathophysiology Laboratory, Instituto Butantan, Av. Vital Brazil, 1500, 05503-900 São Paulo, São Paulo, Brazil; ‡ Department of Pharmaceutical Sciences, Federal University of São Paulo, Rua São Nicolau, 210, 09913030 Diadema, São Paulo, Brazil; § Institute of Chemistry, Federal University of Alfenas (UNIFAL), R. Gabriel Monteiro da Silva, 700, 37130-000 Alfenas, Minas Gerais, Brazil; ∥ Department of Medicine, Federal University of São Paulo (UNIFESP), Av. Dr. Arnaldo, 455, 01246-903 São Paulo, São Paulo, Brazil; ⊥ Centre of Natural Sciences and Humanities, Universidade Federal do ABC, Av. dos Estados, 5001, 09210-580 Santo André, São Paulo, Brazil

## Abstract

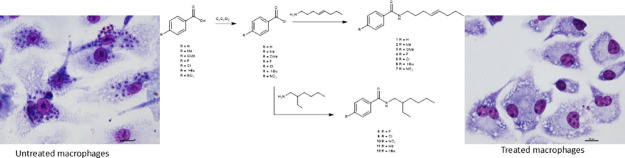

The problems with current strategies to control canine visceral Leishmaniasis (CVL), which include the euthanasia of infected animals, and also the toxicity of the drugs currently used in human treatments for CVL, add urgency to the search for new therapeutic agents. This study aimed to evaluate the activity against *Leishmania (L.) infantum* of 12 amides that are chemically inspired by gibbilimbol B, a bioactive natural product that was initially obtained from *Piper malacophyllum*. Three of these compounds—*N*-(2-ethylhexyl)-4-chlorobenzamide (**9**), *N*-(2-ethylhexyl)-4-nitrobenzamide (**10**), and *N*-(2-ethylhexyl)-4-(*tert*-butyl)benzamide (**12**) —demonstrated activity against the intracellular amastigotes without toxicity to mammalian host cells (CC_50_ > 200 μM); compounds **9**, **10**, and **12** resulted in EC_50_ values of 12.7, 12.2, and 5.1 μM, respectively. *In silico* drug-likeness studies predicted that these compounds would show high levels of gastrointestinal absorption, would be able to penetrate the blood-brain barrier, would show moderate solubility, and would not show unwanted molecular interactions. Due to their promising pharmacological profiles, compounds **9** and **10** were selected for mechanism of action studies (MoA). The MoA studies in *L. (L.) infantum* revealed that neither of the compounds affected the permeabilization of the plasma membrane. Nevertheless, compound **9** induced strong alkalinization of acidocalcisomes, which resulted in a significant and rapid increase in intracellular Ca^2+^ levels, thereby causing the depolarization of the mitochondrial membrane potential and a reduction in the levels of reactive oxygen species (ROS). In contrast, compound **10** induced a gradual increase in intracellular Ca^2+^ levels and a similarly gradual reduction in ROS levels, but it caused neither acidocalcisome alkalinization nor mitochondrial membrane potential depolarization. Finally, the MALDI-TOF/MS assessment of protein alterations in *L. (L.) infantum* treated separately with compounds **9** and **10** revealed changes in mass spectral profiles from both treatments. These results highlight the anti-*L. (L.) infantum* potential of these amides—especially for compounds **9** and **10**—and they suggest that these compounds could be promising candidates for future *in vivo* studies in VL-models.

## 1 Introduction

Canine visceral leishmaniasis (CVL) is a zoonotic systemic infectious disease that is caused by protozoan parasites of the genus *Leishmania.* Several ethical issues make the clinical and prophylactic management of this disease problematic, especially the euthanasia. Relevant problems include the limited efficacy of the current canine vaccine, which shows an efficacy between 68% and 71%,^1^ as well as the variable effectiveness of the euthanasia of infected animals in preventing the spread of infection.^2^ There are several additional justifications of the need to discover new drugs for CVL.

CVL therapy using current human antileishmanial clinical drugs is forbidden by law in Brazil. Additionally, miltefosine is the only drug approved for CVL therapy that contributes to a reduction in the infectivity of *L. (L.) infantum* in dogs. Despite the efficacy of this treatment, the excessive costs of the drug impose restrictions on its use.^3,4^ Consequently, systemic antiparasitic treatments for dogs stand out as important tools for reducing the infectivity of these reservoirs that could affect humans.^5,6^

**Figure 1:**
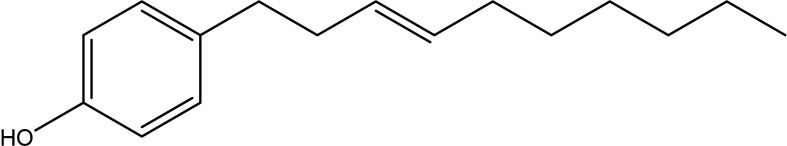
Chemical structure of gibbilimbol B isolated from *P. malacophyllum*.

Compounds based on natural products are considered interesting tools to use in the discovery of bioactive molecules, which contribute to the development of new drugs with antiprotozoal activity.^7,8^ In this context, the alkenyl phenol derivative gibbilimbol B, which was extracted from *Piper malacophyllum* (Figure 1), has exhibited activity against *L. (L.) infantum.*(4,6,9)

Due to the simplicity of the chemical structure of gibbilimbol B, synthetic analogues have been prepared, and these have shown improved drug-likeness profiles and increased potency against *L. (L.) infantum* compared with the natural structure.^10−12^ Other structurally related compounds were prepared with the aim of reducing the toxicity caused by the presence of phenolic group.^11^ Additionally, compared with the related esters, amide derivatives were observed to show clearly improved potency against the parasite.^12^ In the present study, a novel set of amides (compounds **1**–**12**) that were inspired by the natural gibbilimbol B were synthesized and their effects against *L. (L.) infantum* were determined against promastigote and amastigote forms of the parasite. Furthermore, the mechanisms of action of two derivatives were performed.

## 2 Results and Discussion

**Figure 2:**
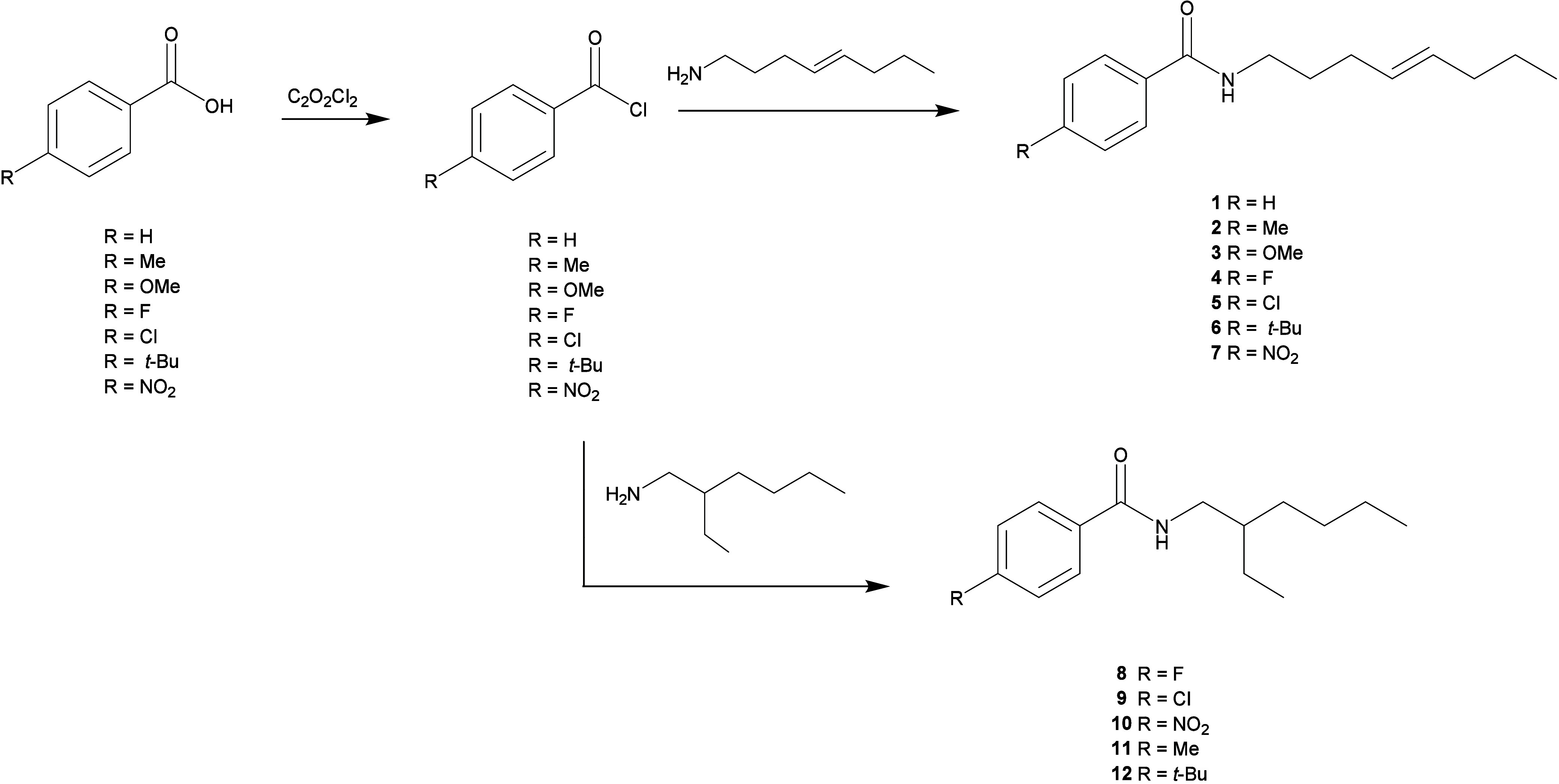
Preparation scheme and structures of amides **1**–**12**.

In the present study, 12 amides (compounds **1**–**12**) (Figure 2) that were inspired by gibbilimbol B were chemically prepared and their in vitro effects were evaluated against amastigote forms of *L. (L.) infantum.*

**Figure 3:**
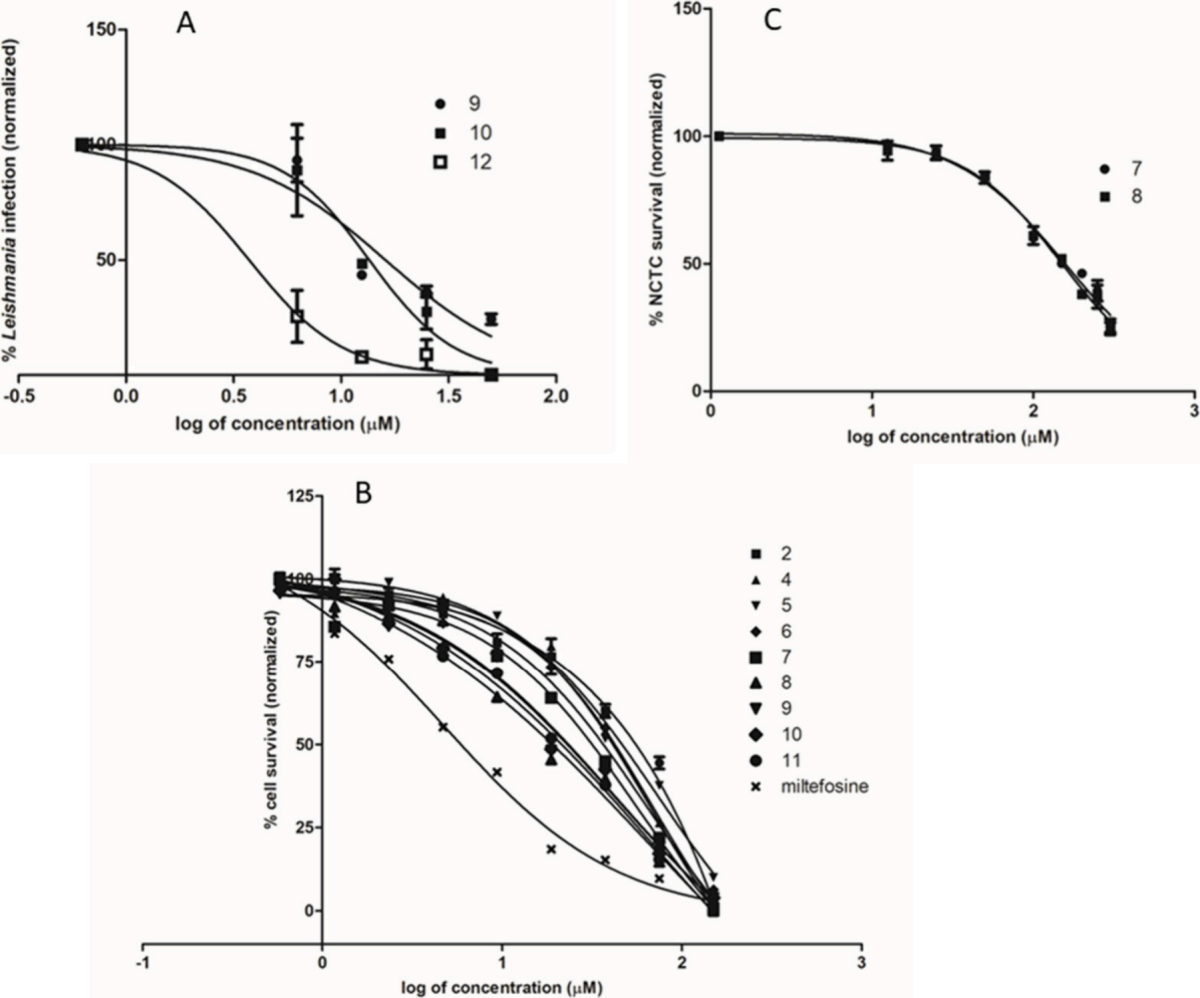
Evaluation of the anti-*L. (L.) infantum* activity (EC_50_) of amides **1**–**12** and miltefosine (standard drug) in amastigotes (A), promastigotes (B), and mammalian cytotoxicity (CC_50_) (C).

As observed in Table 1, except for compounds **1**, **3**, and **12**, all synthesized amides exhibited activity against promastigotes with EC_50_ values between 41.9 and 73.3 μM; these values indicate superior potency compared with that observed for the natural product gibbilimbol B, which showed an EC_50_ value of 99.4 μM (Figure 3).

**Table 1:** Evaluation of *in Vitro* Activity of Natural Gibbilimbol B, Amides **1**–**12** and the Standard Drug Miltefosine against Promastigote and Amastigote Forms *L. (L.) infantum* and NCTC Cells^a^

compound	Ec_50_ Promastigote (μm) ± SD	Ec_50_ Amastigote (μm) ± SD	Cc_50_ Nctc (μm) ± SD	S.I. amastigote
Gibbilimbol B	99.4 ± 2.1	95.1 ± 1.5	>200	>2.1
**1**	>100	>100	>200	-
**2**	66.9 ± 6.4	>100	>200	-
**3**	>150	>100	>200	-
**4**	67.4 ± 1.3	>100	>200	-
**5**	73.3 ± 4.3	>100	>200	-
**6**	66.0 ± 1.0	>100	>200	-
**7**	49.8 ± 4.0	>100	158.2 ± 37.3	-
**8**	64.1 ± 3.5	>100	156.8 ± 1.0	-
**9**	41.9 ± 2.1	12.7 ± 4.9	>200	>15.7
**10**	44.3 ± 0.4	12.2 ± 7.2	>200	>16.4
**11**	66.3 ± 6.0	>100	>200	-
**12**	>150	5.1 ± 1.9	>200	>39.2
**Miltefosine**	5.1 ± 0.6	6.5 ± 3.0	119.7 ± 4.2	18.4

a EC_50_: 50% effective concentration; CC_50_: 50% cytotoxic concentration; SI: selectivity index; SD: standard deviation.

The *ex-vivo* assay using the intracellular amastigotes demonstrated that compounds **9**, **10,** and **12** displayed potent activity against the amastigotes, showing EC_50_ = 12.7, 12.2, and 5.1 μM, respectively; these values were superior to that determined for gibbilimbol B (EC_50_ = 95.1 μM). Compound **12** was the most effective compound in this testing, and it showed similar potency to the standard drug miltefosine (EC_50_ = 6.5 μM). Compounds **1**–**6** and **9**–**12** were no cytotoxic to NCTC cells up to the maximum concentration of 200 μM; in contrast, miltefosine displayed a CC_50_ value of 119.7 μM. Selectivity indexes (SI) for compounds **9**, **10**, and **12**–which were defined as the ratio between CC_50_ against NCTC cells and EC_50_ against *L. (L.) infantum* amastigotes–were determined to be higher than 15.7, 16.4, and 39.2, respectively. These data demonstrate a noncytotoxic profile for compounds **9**, **10**, and **12**, and highlight them as promising hit compounds.

Considering the structures of the amides, it was observed that the compounds containing either an unsaturation at the C-4 position of the side chain with eight carbons (**1**–**7**)—which is similar in structure to the natural product gibbilimbol B–or a side chain with six carbons containing an ethyl group at the C-2 position (**8**–**12**) displayed similar potency against promastigotes. Similarly, the presence of different substituents at the aromatic moiety, including alkyl, methoxy and nitro groups or halogens (except for inactive compounds **1**, **3**, and **12**) caused no important differences in activity against the parasites. However, these substituents affected the potency of the compounds against the intracellular amastigotes, as was observed for compounds **9**, **10**, and **12** that contain a branched side chain. Compound **12** has a *tert*-butyl substituent and displayed the highest potency of all compounds that were tested against the intracellular amastigote of *L. (L.) infantum* (EC_50_ = 5.1 μM) and it showed no mammalian cytotoxicity (CC_50_ > 200 μM; Table 1). According to the Drugs for Neglected Diseases *initiative* (DND*i*) rules,^13^ new anti-*Leishmania* drug candidates are expected to present EC_50_ values less than 10 μM for intracellular amastigotes together with a selectivity index greater than 10. In our study, compounds **9**, **10,** and **12** fulfilled these criteria and can therefore be considered promising hit compounds.

To further assess the potential of these compounds in future *in vivo* studies, different physicochemical characteristics and pharmacokinetic parameters of tested compounds were predicted.^10^ As shown in Table 2, compounds **9**, **10,** and **12**, which showed the required activity and selectivity against amastigotes, were selected to be analyzed in an in silico platform that is used for pharmacokinetic profile prediction.

**Table 2:** *In Silico* Analysis of the Physicochemical, Structural, and ADMET Parameters of Compounds **9**, **10**, and **12**^a^

	compounds		
parameters	9	10	12
Log P	4.54	3.99	5.59
Log S	–4.50	–4.65	–5.68
Log D_7.4_	3.93	3.67	4.36
Rotatable bonds	8	9	9
H-bond acceptors count (N + O)	2	5	2
H-bond donors count (NH + OH)	1	1	1
TPSA (Å^2^)	29.10	72.80	29.10
Lipinski rule-of-five violations	0 (accepted)	0 (accepted)	1 (accepted)
GSK rule	Rejected	Accepted	Rejected
Caco-2 permeability (Log P_app_)	–4.77	–4.85	–4.85
BBB permeability	+	–	–
Plasma protein binding	98.5%	95.3%	98.1%
Pgp substrate	–	–	+
Human intestinal absorption	+	+	+
CYP1A2 inhibitor	+	+	+
CYP2C19 inhibitor	+	–	+
CYP2C9 inhibitor	+	–	+
CYP2D6 inhibitor	+	–	–
CYP3A4 inhibitor	–	+	–
Human liver microsomal stability	+	+	+
Clearance in plasma (mL/min/kg)	5.69	4.92	6.73
*T*_1/2_ (h)	0.63	0.89	0.44
PAINS alerts	0	0	0

a Fsp^3^: Fraction of sp^3^ carbons; TPSA: topological polar surface area; Log P: *n*-octanol/water partition coefficient; Log S: water solubility; Log D_7.4_: distribution coefficient at pH 7.4; Pgp: P-glycoprotein; CYP: cytochrome P450 (and its respective isoforms); PAINS: pan-assay interference compounds; + : yes; −: no.

The results suggested that the compounds that were tested present adequate drug-likeness to be considered bioactive molecules. Analysis of the data describing the physicochemical properties of the compounds determined that compounds **9** and **10** showed balanced lipophilicity (Log P and Log D_7.4_ values) and water solubility (Log S, H-bonds and TPSA values) in addition to passing the Lipinski rule-of-five parameters, whereas compound **12** showed excessive lipophilicity (Log P > 5.0). The water solubility values were also within the limits recommended in the literature (−1 to–5).^14^ The excessive lipophilicity of compound **12** also decreases its pharmacokinetic desirability. It has been shown that highly lipophilic compounds are frequently P-glycoprotein (Pgp) substrates and enzymatic inhibitors, thus increasing their potential for systemic toxicity. As can be noted in Table 2, compound **12** may be subject to efflux by Pgp that could lead to its limited distribution throughout a recipient’s tissues.^15,16^

Moreover, both compounds **9** and **12** appeared to show inhibitory activity against several CYP isoforms. Therefore, the antiparasitic activity results that were obtained suggest that the high lipophilicity of compound **12** may be involved in its ability to reach the intracellular amastigotes. The most suitable drug-like properties were observed for compound **10** due to its lower lipophilicity and improved solubility; this compound also fulfilled the GSK rule parameters (MW < 400 and Log P < 4), suggesting that it has a more favorable ADMET profile than the other compounds.^17^ The predicted pharmacokinetic properties of compound **10**, which included a lower clearance rate compared with the other compounds, identify this compound as the most promising compound in this series. Moreover, none of the compounds that were assessed presented structural alerts for pan-assay interference molecules (PAINS).^18^

**Figure 4:**
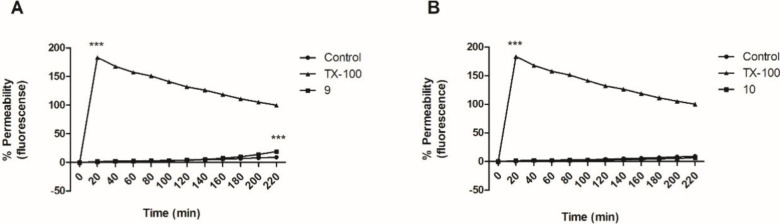
Plasma membrane permeability in *L. (L.) infantum* promastigotes after 4 h of treatment with compound (A) **9** (141.5 μM) or (B) **10** (110.1 μM), using the fluorescent probe Sytox Green (*p* < 0.0001). Negative control: untreated parasites; positive control: Triton X-100 (TX-100).

Due to the demonstrated potency and selectivity of compounds **9** and **10**, in addition to their results from in silico assessments, these two compounds were used in mechanism of action (MoA) studies to investigate their potential lethal toxicity against the parasites; compound 12 was excluded from MoA studies due to its lack of activity against the promastigotes. Changes in the plasma membrane integrity of *Leishmania* spp. can lead to cell death due to pore formation, pH imbalance, and nutrient-transport failure. Initially, the fluorescent probe Sytox Green was employed to analyze the permeability of the plasma membrane. The fluorescence of this dye increases 500-fold after its cell penetration and binding to DNA, thereby demonstrating damage to the plasma membrane.^9,19^ Untreated parasites were used as a negative control for normalization (0% permeabilization) whereas the nonionic surfactant Triton X-100 was used for as positive control (100% permeabilization). As shown in Figure 4, compounds **9** and **10** did not induce permeabilization of the membrane at the first time point after incubation (20 min). In contrast, Triton X-100 demonstrated maximum permeabilization at this 20 min time point, which was greater than that seen in with untreated parasites (*p* < 0.0001). The results from these tests suggest that compounds **9** and **10** do not interfere with membrane permeability.

As previous studies^9,10^ have reported that gibbilimbol B derivatives were involved in membrane perturbation in the parasite, the branched side chain of compounds **9** and **10** may reduce the abilities of these compounds to disrupt plasma membrane integrity, suggesting that they work through an alternative mechanism to kill these parasites membrane integrity, suggesting that they work through an alternative mechanism to kill these parasites kill these parasites membrane integrity, suggesting that they work through an alternative mechanism to kill these parasites.

**Figure 5:**
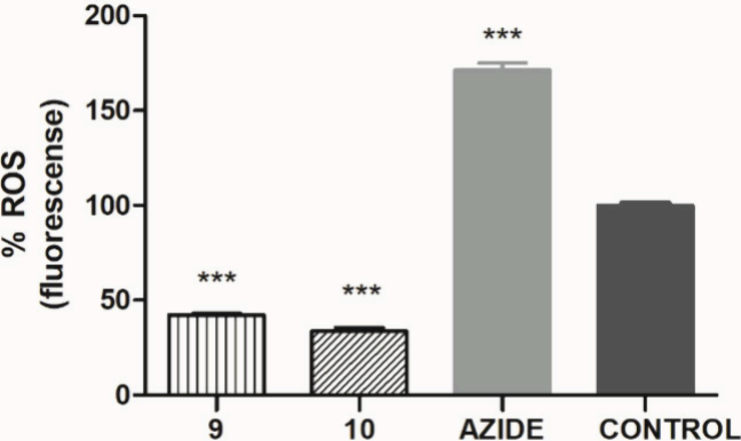
Evaluation of ROS in promastigotes of *L. (L.) infantum* treated with compounds **9** (141.5 μM) or **10** (110.1 μM). H_2_DCFDA dye fluorescence was spectrofluorimetrically measured (excitation and emission at 485 and 520 nm, respectively) after 2 h of incubation. Untreated and treated with NaN_3_ promastigotes were used as negative and positive controls, respectively. ***: *p* < 0.0001.

Previous studies suggested that increased levels of reactive oxygen species (ROS) may be toxic to *Leishmania.*(9,20) Dichlorodihydrofluorescein acetate (H_2_DCFDA) is capable of crossing cell membranes and accumulating in parasites, and thus serves as a marker for detecting free radicals in *L. infantum* promastigotes. Both compounds **9** (at 141.5 μM) and **10** (at 110.1 μM) significantly reduced ROS levels compared with the negative control after 240 min (Figure 5). Sodium azide (positive control) demonstrate the maximum production of ROS whereas untreated parasites were used as a negative control. These results indicated possible effects of compounds **9** and **10** on the mitochondrial respiratory chain, which led to the investigation of their effects on mitochondria.^21^

**Figure 6:**
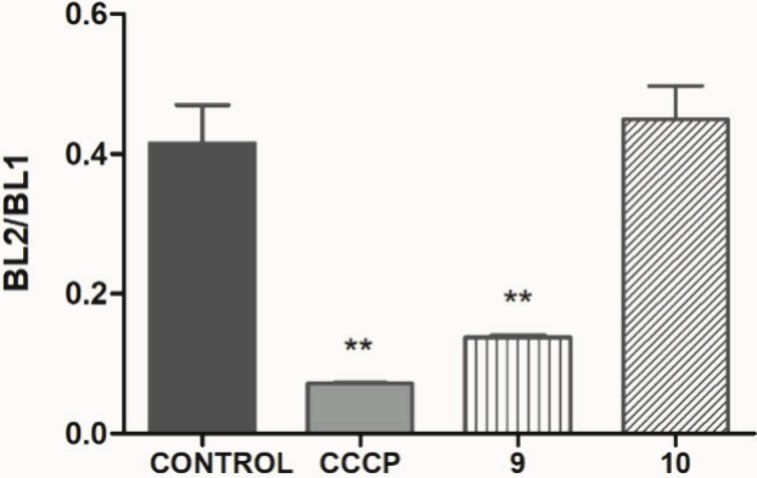
Evaluation of mitochondrial membrane potential in promastigotes of *L. (L.) infantum* treated with compounds **9** (141.5 μM) or **10** (110.1 μM). JC-1 dye fluorescence was measured by flow cytometry (excitation and emission at 488 and 530 nm, respectively) after incubation. Untreated and treated with CCCP (100 μM) promastigotes were used as negative and positive controls, respectively. Fluorescence is reported as the quotient of channels BL2/BL1.**: *p* = 0.0001.

Mitochondria perform several vital functions and play an essential role in maintaining cellular balance, thus making them a potential target for new drugs against *Leishmania* spp. Mitochondria are composed of two interconnected membranes and play crucial roles in energy production (ATP), calcium regulation, and the induction of programmed cell death among other processes.^22,23^ Prolonged changes in mitochondrial membrane potential can lead to irreversible cellular damage.^24^ The mitochondrial membrane potential of *L. (L.) infantum* promastigotes (Figure 6) was evaluated by flow cytometry using JC-1.^25^ After 4h of exposure to compound **9**, a significant depolarization of the mitochondrial membrane was observed compared with the negative control (untreated promastigotes). The compound carbonyl cyanide *m*-chlorophenyl hydrazine (CCCP) was used to induce maximum depolarization (positive control), whereas untreated parasites served as a negative control. Although the difference in results between the untreated control and compound **10** was not statistically significant, it suggested a tendency of compound **10** to hyperpolarize the electric potential of the mitochondria. Both alterations may have contributed to the observed mitochondrial dysfunction and might explain the reduction in ROS levels after treatment that was observed.

**Figure 7:**
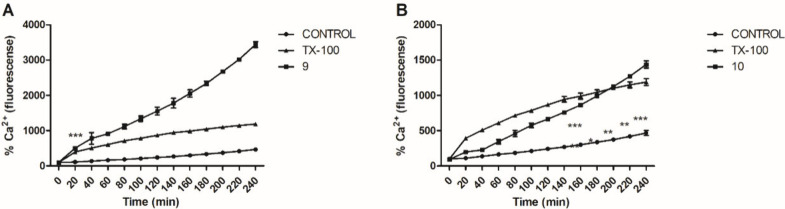
Intracellular Ca^2+^ levels in *L. (L.) infantum* treated with (A) compound **9** (141.5 μM) or (B) compound **10** (110.1 μM). Results were obtained using the Fluo-4 AM dye (excitation and emission at 485 and 535 nm, respectively). Untreated and treated with Triton X-100 (0.5% v/v) parasites were used as negative and positive controls, respectively. Fluorescence is represented as a percentage relative to the untreated control at 0 min (100%).***p < 0.0001.

Compounds that affect intracellular Ca^2+^ balance in *Leishmania* may cause changes in the mechanism of transport of these ions in trypanosomatids.^26^ In *Leishmania* spp., Ca^2+^ regulation involves the endoplasmic reticulum, the mitochondria, and the acidocalcisomes;^27,28^ increases in this intracellular ion can trigger processes such as apoptosis that led to the death of the parasite.^29^ The Fura-2 AM marker was used to measure intracellular Ca^2+^ levels in *Leishmania (L.) infantum* promastigotes. Fura-2 has a high affinity for Ca^2+^, which results in the formation of fluorescent intracellular complexes that are proportional to intracellular Ca^2+^ levels.^30^ Both compounds **9** (Figure 7A) and **10** (Figure 7B) increased the intracellular Ca^2+^ concentration in *L. (L.) infantum*, and showed significant differences compared with negative (untreated parasites) and positive controls. (Triton X-100) at all monitored times. Compound **9** rapidly increased Ca^2+^ levels, which approached those of the positive control within 20 min. Compound **10** induced similar levels of fluorescence to those induced by compound **9** but at a slower rate, as these levels were observed after 200 min of incubation.

The extravasation of Ca^2+^ ions with consequent accumulation in the cytoplasm can contribute to mitochondrial imbalance.^30^ Ca^2+^ transport mechanisms in trypanosomatids and eukaryotic cells involve proteins such as MCU, VDAC1, and CHX, which influence the movement of Ca^2+^ in and out of mitochondria.^31,32^

**Figure 8:**
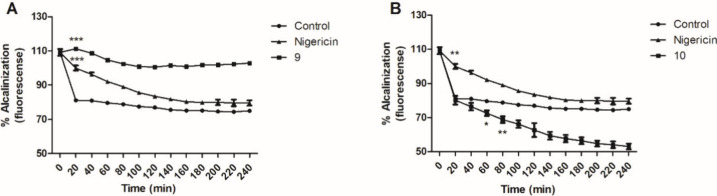
Acidocalcisome alkalization in *L. (L.) infantum* treated with compounds **9** (141.5 μM - A) or **10** (110.1 μM - B). Results were obtained using acridine orange dye (excitation and emission at 485 and 535 nm, respectively). Untreated and treated with nigericin parasites were used as negative and positive controls, respectively. Fluorescence is represented as a percentage of the fluorescence in the positive control with nigericin at 20 min (100%). ***p = 0.003, **p = 0.0021, **p = 0.05.

Acidocalcisomes are intracellular organelles that are characterized by acidity and high calcium concentrations due to the proton and calcium pumps.^33^ In trypanosomatids, these organelles are rich in short-chain polyphosphates and occupy a small proportion (1 to 2%) of each cell’s volume.^34^ In these parasites, the acidocalcisomes are responsible for capturing and storing calcium in large quantities, which is essential for cellular functioning.^35^ In our study, we investigated possible changes resulting from treatment with compounds in acidocalcisomes, especially concerning intracellular calcium. Acridine orange was used as a fluorescent probe to evaluate changes in the acidocalcisomes of *L. (L.) infantum* promastigotes. Alkalinization of these organelles results in the release of the probe, altering the fluorescence.^36^ A short incubation (20 min) with compound **9** (Figure 8A) induced an increase in the fluorescence levels as a result of the alkalinization of the acidocalcisomes. This result corroborates the high calcium levels that we detected after incubation with compound **9** and suggest that acidocalcisomes are a source of this calcium. In contrast, incubation with compound **10** (Figure 8B) caused the progressive and continuous acidification of these organelles over time, with a statistically significant difference compared with the negative control observed from 60 min onward; this effect may have resulted from deficits in mitochondrial functioning and the low levels of ROS that were observed. These effects were compared with those of the positive (nigericin) and negative (untreated parasites) controls.

**Figure 9:**
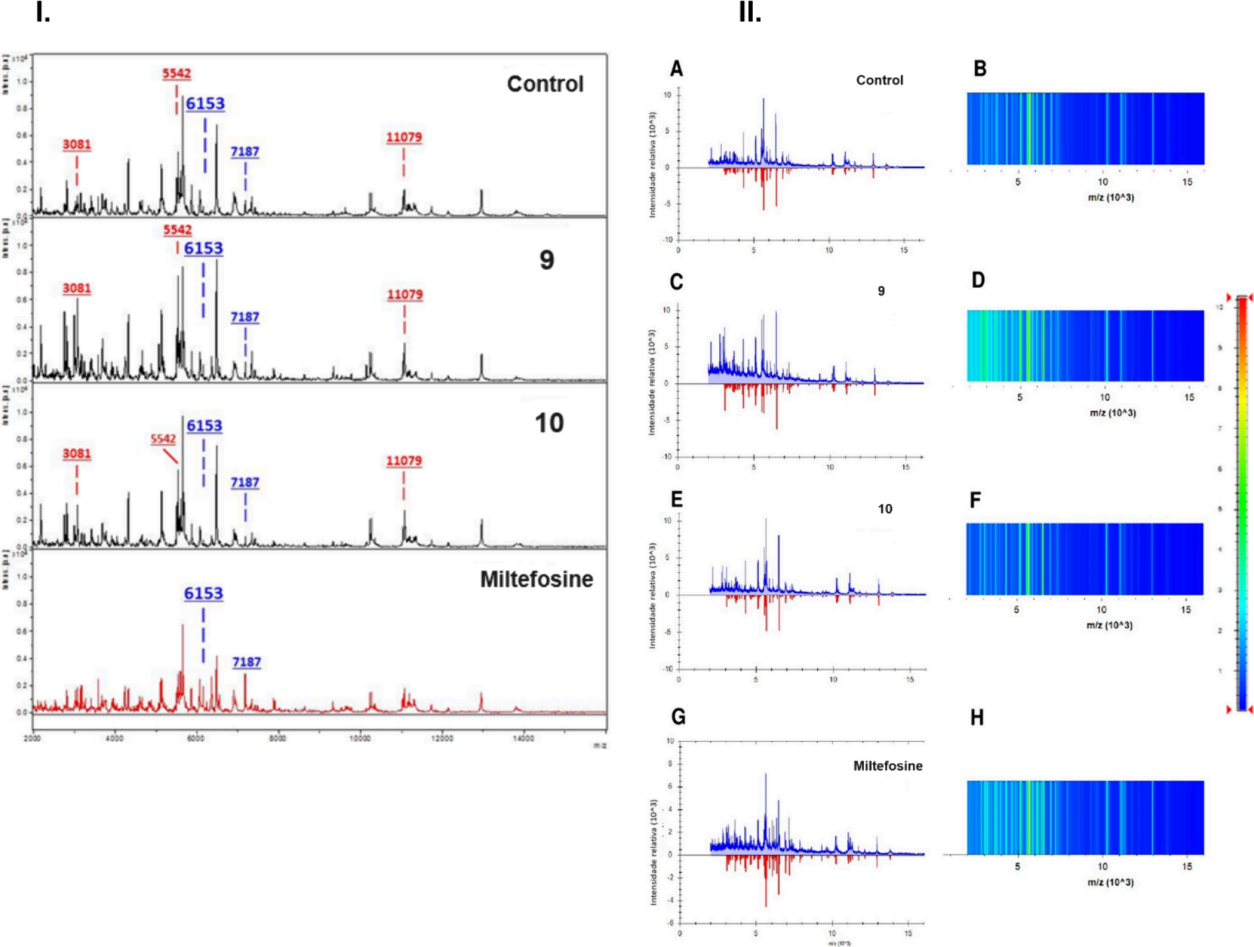
Protein-mass profile of promastigotes of *L. (L.) infantum* from MALDI-TOF/MS. Panel **I**. Promastigotes were incubated with compound **9** (141.5 μM), compound **10** (110.1 μM). Miltefosine was used as positive control whereas untreated parasites were used as a negative control. The peaks observed at *m*/*z* 6,153 and *m*/*z* 7,187 (blue) allow the identification of the *Leishmania* subgenus. Increases in peak intensity resulting from the incubation of promastigotes with compound **9** or **10** can be observed at *m*/*z* 3,081, 5,542, and 11,079 (red). Panel **II**. A schematic gel showing mass spectra in the *m*/*z* 2,000–20,000 range (B, D, F, H); raw spectra are shown in blue (A, C, E, G), whereas spectra generated after normalization, smoothing, and baseline subtraction are shown in red. (A) and (B), results from untreated promastigotes (negative control); (C) and (D); results from promastigotes incubated with compound **9**; (E) and (F), results from promastigotes incubated with compound **10**; and (G) and (H) results from promastigotes incubated with miltefosine (standard treatment drug and positive control).

Finally, the protein variations of *Leishmania* after treatment with compounds **9** and **10** were investigated using MALDI-TOF/MS analysis.^37,38^ It was observed that incubation of *Leishmania* with compound **9** or **10** for 24 h increased the intensity of the spectral peaks compared with the untreated parasites (negative control), as seen in Figure 9. The differences in spectral profiles that were induced by compounds **9** and **10** compared with miltefosine suggest that future combination therapy studies would be worthwhile. Results describing the ribosomal and structural proteins from the MALDI-TOF/MS fingerprints of treated *L. (L.) infantum*(39) suggest that these compounds could affect the protein metabolism of the parasite.

## 3 Conclusions

Assessment of the antileishmanial activities of 12 chemically related amides against *L. (L.) infantum* resulted in the identification of three compounds (**9**, **10**, and **12**) that showed potent activity against the intracellular amastigotes of *Leishmania*, with no toxicity against mammalian cells. Subsequently, an in silico analysis of the compounds’ drug-likeness profiles identified compound **10** as the most promising compound based on its pharmacokinetic profile. MoA studies showed that compound **9** induced an intense depolarization of the mitochondrial membrane, a reduction in ROS levels, the alkalinization of acidocalcisomes, and the elevation of intracellular Ca^2+^; in contrast, compound **10** showed a less intense and more gradual effect on Ca^2+^ release and it reduced ROS levels without affecting the mitochondrial membrane potential. Finally, mass spectrometry confirmed the presence of alterations at the protein level, providing additional evidence of harmful cellular effects that might have contributed to the death of the parasite. Future *in vivo* pharmacokinetic and efficacy studies in VL-models could demonstrate the applicability of these bioactive amides as candidates for the development of new drugs for CVL treatment.

## 4 Materials and Methods

### 4.1 General Procedures

Silica gel-G and silica gel 60 (0.040–0.063 mm) were used for thin-layer (TLC) and column (CC) chromatography, respectively. NMR spectra were recorded on a Bruker AC-300 spectrometer (300 MHz for ^1^H and 75 MHz for ^13^C NMR, respectively) using CDCl_3_ as a solvent and TMS as an internal standard. ESI-HRMS spectra (positive mode) were measured on a Bruker Daltonics q-TOF Maxis 3G spectrometer. IR spectra were recorded on a Shimadzu FTIR-Affinity-1 or Thermo Scientific Nicolet-iS50 instrument. Absorbance-, fluorescence- and luminescence-based assays were performed on a FilterMax F5Multi-Mode Microplate Reader (Molecular Devices) or a Atunne NxT flow cytometer (Thermo Fisher Scientific).

### 4.2 Preparation of Compounds **1**–**12**

To 12 different round bottomed flasks was added 1.56 mmol (1.3 equiv) of each acyl chloride that had been prepared from respective benzoic acid and C_2_O_2_Cl_2_,^40^ which was dissolved in CH_2_Cl_2_ (10 mL) with NaOH (2 mol/L–4 mL). The temperature of each reaction was maintained at 0 °C and 1.2 mmol (1 equiv) of (*E*)-oct-4-en-1-amine (for compounds **1**–**7**) or 2-ethylhexan-1-amine (for compounds **8**–**12**), dissolved in CH_2_Cl_2_ (5 mL) were slowly added. The obtained solution was kept under stirring until the starting materials had been consumed, which was monitored by TLC (hexane:EtOAc 7:3 v/v) and staining with alcoholic ninhydrin (0.2%, w/v). The crude product was sequentially treated with H_2_O, NaOH (10%, w/v), HCl (10%, v/v), and brine. The residue was dried over Na_2_SO_4_, filtered, and the solvent was eliminated under reduced pressure. Each product was purified over silica gel column eluted with hexane:EtOAc (9:1 to 7:2 v/v) to afford amides **1**–**12**.

#### 4.2.1 (*E*)-*N*-(Oct-4-en-1-yl)benzamide (**1**)

Colorless oil. IR, ν_max_/cm^–1^: 3309, 2927, 1627, 973. ^1^H NMR, δ/ppm: 7.76–7.73 (m), 7.52–7.39 (m), 6.19 (s), 5.52–5.36 (m), 3.45 (q, *J* = 6.0 Hz), 2.13–2.06 (m), 1.99–1.93 (m), 1.73–1.63 (quint, *J* = 7.4 Hz), 1.35 (sext, *J* = 7.4 Hz), 0.87 (t, *J* = 7.3 Hz). ^13^C NMR, δ/ppm: 167.5, 134.8, 131.4, 131.3, 129.2, 128.6, 126.8, 39.7, 34.7, 30.2, 29.4, 22.6, and 13.7. ESI-HRMS *m*/*z* 232.1698 [M + H]^+^ (calculated for C_15_H_22_NO, 232.1695).

#### 4.2.2 (*E*)-4-Methyl-*N*-(oct-4-en-1-yl)benzamide (**2**)

Yellow oil. IR, ν_max_/cm^–1^: 3314, 2926, 1633, 1541, 1505, 965. ^1^H NMR, δ/ppm: 7.68 (d, *J* = 8.7 Hz), 7.42 (d, *J* = 8.7 Hz), 6.29 (s), 5.51–5.36 (m), 3.44 (q, *J* = 6.9 Hz), 2.09 (q, *J* = 7.0 Hz), 1.95 (q, *J* = 7.0 Hz), 1.67 (quint, *J* = 7.4 Hz), 1.41–1.29 (m), 0.87 (t, *J* = 7.3 Hz). ^13^C NMR, δ/ppm: 167.4, 141.7, 132.0, 131.3, 129.2, 129.2, 126.8, 39.7, 34.7, 30.2, 29.4, 22.6, 21.4, and 13.7. ESI-HRMS *m*/*z* 246.1848 [M + H]^+^ (calculated for C_16_H_24_NO, 246.1852).

#### 4.2.3 (*E*)-4-Methoxy-*N*-(oct-4-en-1-yl)benzamide (**3**)

Colorless oil. IR, ν_max_/cm^–1^: 3309, 2927, 1627, 973. ^1^H NMR, δ/ppm: 7.71 (d, *J* = 8.7 Hz), 6.90 (d, *J* = 8.7 Hz), 6.22 (s), 5.50–5.35 (m), 3.42 (q, *J* = 6.9 Hz), 2.07 (q, *J* = 7.1 Hz), 1.94 (q, *J* = 7.4 Hz), 1.65 (quint, *J* = 7.4 Hz), 1.34 (sex, *J* = 7.4 Hz), 0.86 (t, *J* = 7.3 Hz). ^13^C NMR, δ/ppm: 167.1, 162.1, 131.4, 129.4, 128.7, 127.2, 113.8, 55.5, 39.8, 34.8, 30.3, 29.6, 22.7, and 13.8. ESI-HRMS *m*/*z* 262.1804 [M + H]^+^ (calculated for C_16_H_24_NO_2_, 262.1801).

#### 4.2.4 *(*E)-4-Fluoro-N-(oct-4-en-1-yl)benzamide (**4**)

Yellow oil. IR, ν_max_/cm^–1^: 3296, 2928, 1627, 973. ^1^H NMR, δ/ppm: 7.71 (d, *J* = 8.7 Hz), 6.90 (d, *J* = 8.7 Hz), 6.22 (s), 5.50–5.35 (m), 3.42 (q, *J* = 6.9 Hz), 2.07 (q, *J* = 7.1 Hz), 1.94 (q, *J* = 7.4 Hz), 1.65 (quint, *J* = 7.4 Hz), 1.34 (sex, *J* = 7.4 Hz), 0.86 (t, *J* = 7.3 Hz). ^13^C NMR, δ/ppm: 166.5, 163.1, 131.3, 129.3, 129.2, 115.7, 39.9, 34.8, 30.3, 29.5, 22.8, and 13.8. ESI-HRMS *m*/*z* 250.1604 [M + H]^+^ (calculated for C_15_H_21_FNO, 250.1601).

#### 4.2.5 *(*E)-4-Chloro-*N*-(oct-4-en-1-yl)benzamides (**5***)*

White solid. IR, ν_max_/cm^–1^: 3325, 2924, 1631, and 976. ^1^H NMR, δ/ppm: δ 7.68 (d, *J* = 8.7 Hz), 7.39 (d, *J* = 8.7 Hz), 6.15 (s), 5.52–5.36 (m), 3.44 (q, *J* = 6.9 Hz), 2.09 (q, *J* = 7.2 Hz), 1.95 (q, *J* = 7.1 Hz), 1.67 (quint, *J* = 7.4 Hz), 1.35 (sex, *J* = 7.5 Hz), 0.87 (t, *J* = 7.3 Hz). ^13^C NMR, δ/ppm: 165.5, 137.7, 133.3, 131.6, 129.2, 128.9, 128.4, 40.0, 34.8, 30.3, 29.4, 22.8, 13.8. ESI-HRMS *m*/*z* 266.1303 [M + H]^+^ (calculated for C_15_H_21_ClNO, 266.1306).

#### 4.2.6 (*E*)*-*4*-*(*tert*-butyl)-*N*-(oct-4-en-1-yl)benzamide (**6**)

Yellow oil. IR, ν_max_/cm^–1^: 3314, 2926, 1633, and 965. ^1^H NMR, δ/ppm: 7.68 (d, *J* = 8.7 Hz), 7.42 (d, *J* = 8.7 Hz), 6.29 (s), 5.51–5.36 (m), 3.44 (q, *J* = 6.9 Hz), 2.09 (q, *J* = 7.0 Hz), 1.95 (q, *J* = 7.0 Hz), 1.67 (quint, *J* = 7.4 Hz), 1.41–1.29 (m), 0.87 (t, *J* = 7.3 Hz). ^13^C NMR, δ/ppm: 167.5, 154.9, 132.1, 131.5, 129.4, 126.8, 125.6, 39.7, 35.0, 34.8, 31.3, 30.3, 29.6, 22.8, and 13.8. ESI-HRMS *m*/*z* 288.2319 [M + H]^+^ (calculated for C_19_H_30_NO, 288.2321).

#### 4.2.7 (*E*)-4-Nitro-*N*-(oct-4-en-1-yl)benzamide (**7**)

White crystals. IR, ν_max_/cm^–1^: 3292, 2923, 1634, 1343, 1315, and 976. ^1^H NMR, δ/ppm: 8.27 (d, *J* = 8.9 Hz), 7.90 (d, *J* = 8.9 Hz), 6.30 (s), 5.52–5.36 (m), 3.48 (q, *J* = 6.9 Hz), 2.11 (q, *J* = 7.2 Hz), 1.95 (q, *J* = 7.1 Hz), 1.70 (quint, *J* = 7.1 Hz), 1.35 (sex, *J* = 7.4 Hz), 0.87 (t, *J* = 7.3 Hz). ^13^C NMR, δ/ppm: 165.6, 149.6, 140.5, 131.8, 129.1, 128.2, 124.0, 40.2, 34.8, 30.2, 29.3, 22.8, and 13.8. ESI-HRMS *m*/*z* 277.1547 [M + H]^+^ (calculated for C_15_H_21_N_2_O_3_, 277.1546).

#### 4.2.8 *N*-(2-Ethylhexyl)-4-fluorobenzamide (**8**)

Colorless oil. IR, ν_max_/cm^–1^: 3311, 2959, 2926, 2862, 1633, 1502, 1234, 849, and 768. ^1^H NMR, δ/ppm: 7.78–7.72 (m), 7.13–7.06 (m), 6.05 (s), 3.38 (dt, *J* = 6.0 and 1.8 Hz), 1.55 (quint, *J* = 6.4 Hz), 1.42–1.31 (m), 0.94–0.86 (m). ^13^C NMR, δ/ppm: 166.5, 131.1, 129.1, 115.5, 43.0, 39.5, 31.1, 28.9, 24.4, 23.0, 14.1, 10.9. ESI-HRMS *m*/*z* 252.1762 [M + H]^+^ (calculated for C_15_H_23_FNO, 252.1758).

#### 4.2.9 *N*-(2-Ethylhexyl)-4-chlorobenzamide (**9**)

White solid. IR, ν_max_/cm^–1^: 3254, 2955, 2928, 2861, 1629, 1544, 1486, 1319, 1090, 844, 716, and 403. ^1^H NMR, δ/ppm: 7.68 (d, *J* = 8.6 Hz), 7.38 (d, *J* = 8.6 Hz), 6.10 (s), 3.37 (dt, *J* = 6.0 and 1.6 Hz), 1.55 (quint, *J* = 5.7 Hz), 1.41–1.29 (m), 0.94–0.86 (m). ^13^C NMR, δ/ppm: 166.6, 137.5, 133.3, 128.8, 128.3, 43.0, 39.5, 31.1, 28.9, 24.3, 23.0, 14.1, and 10.9. ESI-HRMS *m*/*z* 268.1459 [M + H]^+^ (calculated for C_15_H_23_ClNO, 268.1462).

#### 4.2.10 *N*-(2-Ethylhexyl)-4-nitrobenzamide (**10**)

Colorless oil. IR, ν_max_/cm^–1^: 3032, 2959, 2926, 2861, 1642, 1529, 1340, 862, and 718. ^1^H NMR, δ/ppm: 8.27 (d, *J* = 8.7 Hz), 7.90 (d, *J* = 8.7 Hz), 6.22 (s), 3.41 (dt, *J* = 13.5 and 6.0 Hz), 1.58 (quint, *J* = 6.4 Hz), 1.43–1.31 (m), 0.95–0.89 (m). ^13^C NMR, δ/ppm: 165.6, 149.5, 140.5, 128.0, 123.8, 43.3, 39.4, 31.1, 28.9, 24.3, 23.0, 14.1, and 10.9. ESI-HRMS *m*/*z* 279.1707 [M + H]^+^ (calculated for C_15_H_23_N_2_O_3_, 279.1703).

#### 4.2.11 *N*-(2-Ethylhexyl)-4-methylbenzamide (**11**)

Pale solid. IR, ν_max_/cm^–1^: 3331, 2953, 2920, 2861, 1628, 1537, 1503, 841, and 758. ^1^H NMR, δ/ppm: 7.64 (d, *J* = 8.1 Hz), 7.21 (d, *J* = 8.1 Hz), 6.10 (s), 3.37 (dt, *J* = 6.0 and 1.6 Hz), 2.38 (s), 1.55 (quint, *J* = 6.0 Hz), 1.41–1.30 (m), 0.94–0.86 (m). ^13^C NMR, δ/ppm: 167.6, 141.7, 132.1, 129.2, 126.8, 42.9, 39.5, 31.1, 28.9, 24.4, 23.0, 21.4, 14.1, and 10.9. ESI-HRMS *m*/*z* 248.2011 [M + H]^+^ (calculated for C_16_H_26_NO, 248.2008).

#### 4.2.12 *N*-(2-Ethylhexyl)-4-(*tert*-butyl)benzamide (**12**)

Yellowish oil. IR, ν_max_/cm^–1^: 3312, 2957, 2928, 2869, 1632, 1542, 854, and 777. ^1^H NMR, δ/ppm: 7.73 (d, *J* = 8.5 Hz), 7.43 (m, *J* = 8.5 Hz), 6.01 (s), 3.38 (dt, *J* = 6.0 and 1.0 Hz), 1.56–1.53 (m), 1.41–1.32 (m), 0.94–0.89 (m). ^13^C NMR, δ/ppm: 167.6, 154.8, 132.1, 126.6, 125.5, 42.8, 39.5, 34.9, 31.2, 28.9, 24.4, 23.0, 14.1, and 11.0. ESI-HRMS *m*/*z* 290.2480 [M + H]^+^ (calculated for C_19_H_32_NO, 290.2478).

### 4.3 Animals, Parasites and Mammalian Cell Maintenance

Information concerning the animal facilities, parasites and mammalian cell maintenance have previously been reported.^8−12^ All procedures that were performed were approved by the Animal Care and Use Committee from the Instituto Adolfo Lutz - Secretary of Health of São Paulo State (Project CTC 72-J/2017).

### 4.4 Evaluation of in Vitro Anti-*L. infantum* Activity

Evaluation of *in vitro* activity against promastigote and amastigote forms of *L. (L.) infantum* was performed as previously reported assays.^40−43^

### 4.5 Evaluation of *in Vitro* Mammalian Toxicity

Evaluation of in vitro mammalian toxicity against NCTC cells was performed as previously described.^40^ The selectivity index was determined using the following equation: CC_50_ against NCTC cells/EC_50_ against amastigotes.^44^

### 4.6 Computational Analysis of Physicochemical Properties and Pharmacokinetic Parameters

To evaluate the pharmacokinetic potential and drug-likeness of the most active compounds, compounds **9**, **10,** and **12** were analyzed using ADMETlab 3.0 software.^45^ This software allows the calculation of various physicochemical, medicinal chemistry, pharmacokinetic, and toxicity parameters. The selected properties were tabulated and presented in Table 2.

### 4.7 Evaluation of the MoA in *L*. (*L*.) *infantum*

#### 4.7.1 Determination of Plasma Membrane Integrity

Assays for determination of plasma membrane integrity of *L. (L.) infantum* were conducted as previously reported.^46^ Briefly, compounds **9** (141.5 μM) and **10** (110.1 μM) were added to different wells and the resulting fluorescence was measured using a fluorimetric microplate reader (FilterMax F5Multi-Mode, Molecular Devices). Triton X-100 (0.5%, v/v) and untreated parasites were used as positive negative controls, respectively.^47^

#### 4.7.2 Mitochondrial Membrane Electric Potential (ΔΨm) Analysis

Analysis of mitochondrial membrane electric potential (ΔΨm) in *L. (L.) infantum* were conducted as previously reported.^38^ Briefly, promastigotes were treated with compounds **9** (141.5 μM) or **10** (110.1 μM) in HBSS+Glu at 24 °C using JC-1 dye (Molecular Probes). Fluorescence was measured with excitation and emission filters at 488 and 530 nm (BL-1)/574 nm (BL-2), respectively. Untreated and treated parasite (CCCP - 100 μM) were used as negative and positive controls, respectively.

#### 4.7.3 Measurement of ROS Generation

Measurements of ROS were conducted as previously reported.^48^ Briefly, promastigotes were treated with compounds **9** (141.5 μM) or **10** (110.1 μM) in HBSS+Glu at 24 °C using H_2_DCFDA (Molecular Probes). Fluorescence was measured using a fluorimetric microplate reader (FilterMax F5Multi-Mode, Molecular Devices) with excitation and emission filters at 485 and 520 nm, respectively.^48^ Untreated and treated parasites (H_2_O_2_ - 40 μM) were used as negative and positive controls, respectively.

#### 4.7.4 Measurement of Intracellular Calcium Levels (Ca^2+^)

Measurements of intracellular Ca^2+^ levels were conducted as previously reported.^49^ Briefly, promastigotes were pretreated with Fura-2 AM (Molecular Probes) in PBS, washed and treated with compounds **9** (141.5 μM) or **10** (110.1 μM). Fluorescence was measured using a microplate reader (FilterMax F5Multi-Mode, Molecular Devices) with excitation and emission at 360 and 500 nm, respectively. Untreated and treated parasites (0.5% Triton X-100) were used as negative and positive controls, respectively.

#### 4.7.5 Acidocalcisomes

Alterations in acidocalcisomes were conducted as previously reported.^36^ Briefly, promastigotes were stained with acridine orange (4 μM) in PBS and the parasites were incubated with compounds **9** (141.5 μM) or **10** (110.1 μM). The fluorescence was measured in a spectrofluorimeter with excitation an emission at 535 and 485 nm, respectively. Untreated and treated parasites (nigericin -4 μM) were used as negative and positive controls, respectively.

#### 4.7.6 Parasite Protein Spectral Profiling (MALDI-TOF MS)

MALDI-TOF/MS was used to investigate protein changes in stationary-phase *L. (L.) infantum* promastigotes that were induced by treatment with compound **9** (141.5 μM), compound **10** (110.1 μM), or the standard drug miltefosine. Proteins were extracted from the treated promastigotes and were prepared for analysis on a BrukerAutoflex III MALDI-TOF/MS mass spectrometer (Bruker Daltonics, Bremen, Germany). The mass range was calibrated between *m*/*z* 2,000 and 20,000. A total of 500 random laser shots were collected for each spectrum. Analyzes were conducted using MaldiBiotyper 3.1 software and only peaks with a signal-to-noise ratio greater than three were considered.^36^

### 4.8 Statistical Analysis

The CC_50_ and EC_50_ values were calculated using sigmoidal dose–response curves using Graph Pad Prism 5.0 software. One-way ANOVA testing with Tukey’s multiple comparison tests were used for evaluation of the statistical significance (*p*-value) of differences between the samples, which were tested in duplicate and all assays were repeated at least twice.

## Supporting Information Available

NMR and ESI-HRMS spectra of compounds **1**–**12** (PDF)
