# Supplementary material for: Toward New Therapeutics for Visceral Leishmaniasis: Efficacy and Mechanism of Action of Amides Inspired by Gibbilimbol B
Source: ACS Omega. 2024 Oct 8;9(44):44385–95. doi: 10.1021/acsomega.4c05510 (PMC11541474; doi:10.1021/acsomega.4c05510)

Supplementary Information

Toward New Therapeutics for Visceral Leishmaniasis: Efficacy and Mechanism of Action of Amides Inspired by Gibbilimbol B

Fabio Navarro Baltazar[a‡], Maiara Amaral[a‡], Maiara Maria Romanelli[a], Erica Valadares de Castro Levatti[a], Fernanda Fonseca Ramos[a,b], Luiz Luiz Paulo Melchior Oliveira Leão[c], Daniela Aparecida Chagas-Paula[c], Marisi Gomes Soares[c], Danielle Ferreira Ferreira Dias[c], Cecilia M. S. Q. Aranha[d], João Paulo dos Santos Fernandes[b], Joao Henrique Ghilardi Lago[e*], Andre Gustavo Tempone[a*]

[a] Pathophysiology Laboratory, Instituto Butantan, Av. Vital Brazil, 1500, Butantan, 05503-900, São Paulo - SP, Brazil.

[b] Department of Pharmaceutical Sciences, Federal University of São Paulo. Rua São Nicolau, 210, 2º andar, Centro, 09913030 Diadema - SP, Brazil

[c] Institute of Chemistry, Federal University of Alfenas (UNIFAL). R. Gabriel Monteiro da Silva, 700, Centro, 37130-000 Alfenas - MG, Brazil

[d] Department of Medicine, Federal University of São Paulo (UNIFESP). Av. Dr. Arnaldo, 455 - Cerqueira César, 01246-903, São Paulo - SP, Brazil

[e] Centre of Natural Sciences and Humanities, Universidade Federal do ABC. Av. dos Estados, 5001, Bairro Bangu, 09210-580, Santo André – SP, Brazil.

‡ These authors contributed equally to the work.

Corresponding authors:

Andre Gustavo Tempone, andre.tempone@butantan.gov.br

Av. Vital Brazil, 1500, Butantan, 05503-900, São Paulo - SP, Brazil.

João Henrique G. Lago, joao.lago@ufabc.edu.br

Av. dos Estados, 5001, Bairro Bangu, 09210-580, Santo André – SP, Brazil.

Figure S1. ^{1}H NMR spectrum (300 MHz, $CDCl_3$) of compound **1**

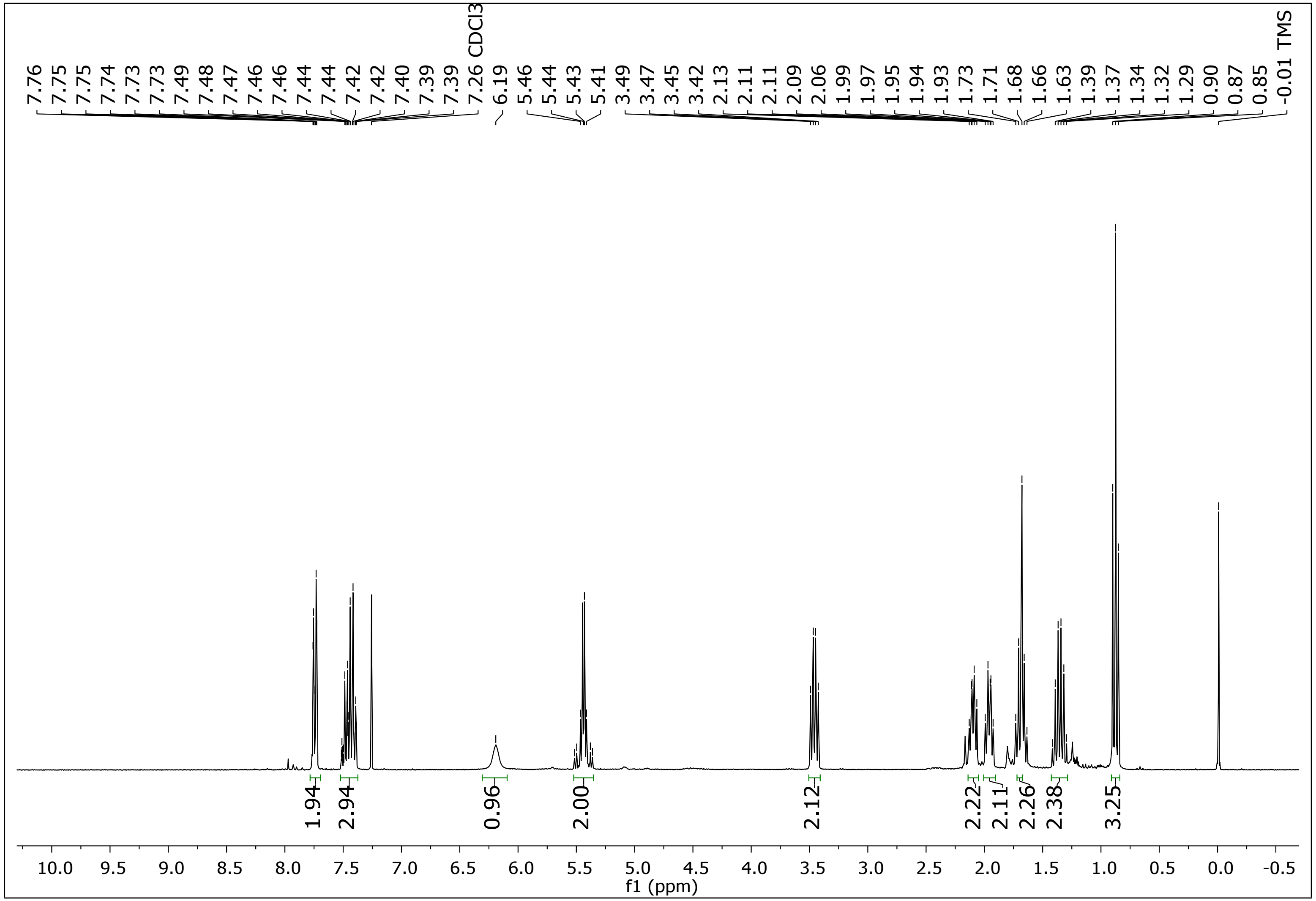

Figure S2. ^{13}C NMR spectrum (75 MHz, $CDCl_3$) of compound **1**

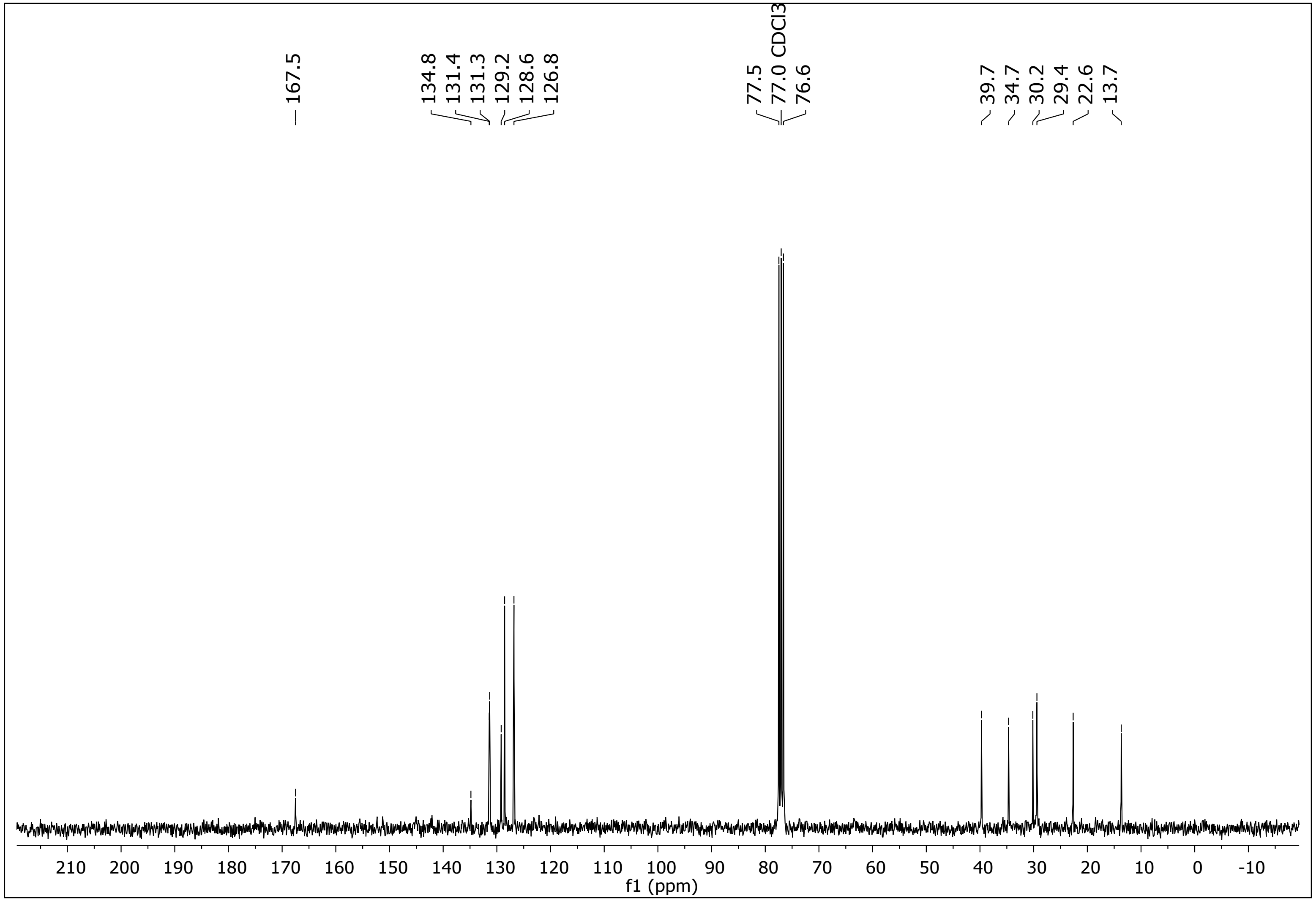

Figure S3. HRMS spectrum of compound **1**

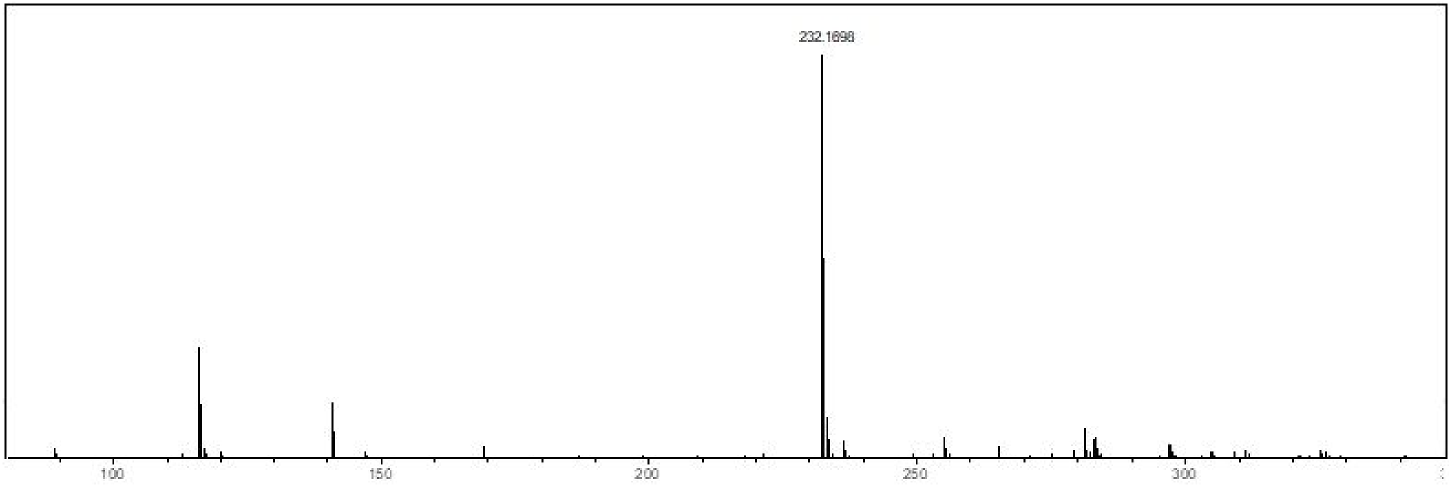

Figure S4. ^{1}H NMR spectrum (300 MHz, $CDCl_3$) of compound **2**

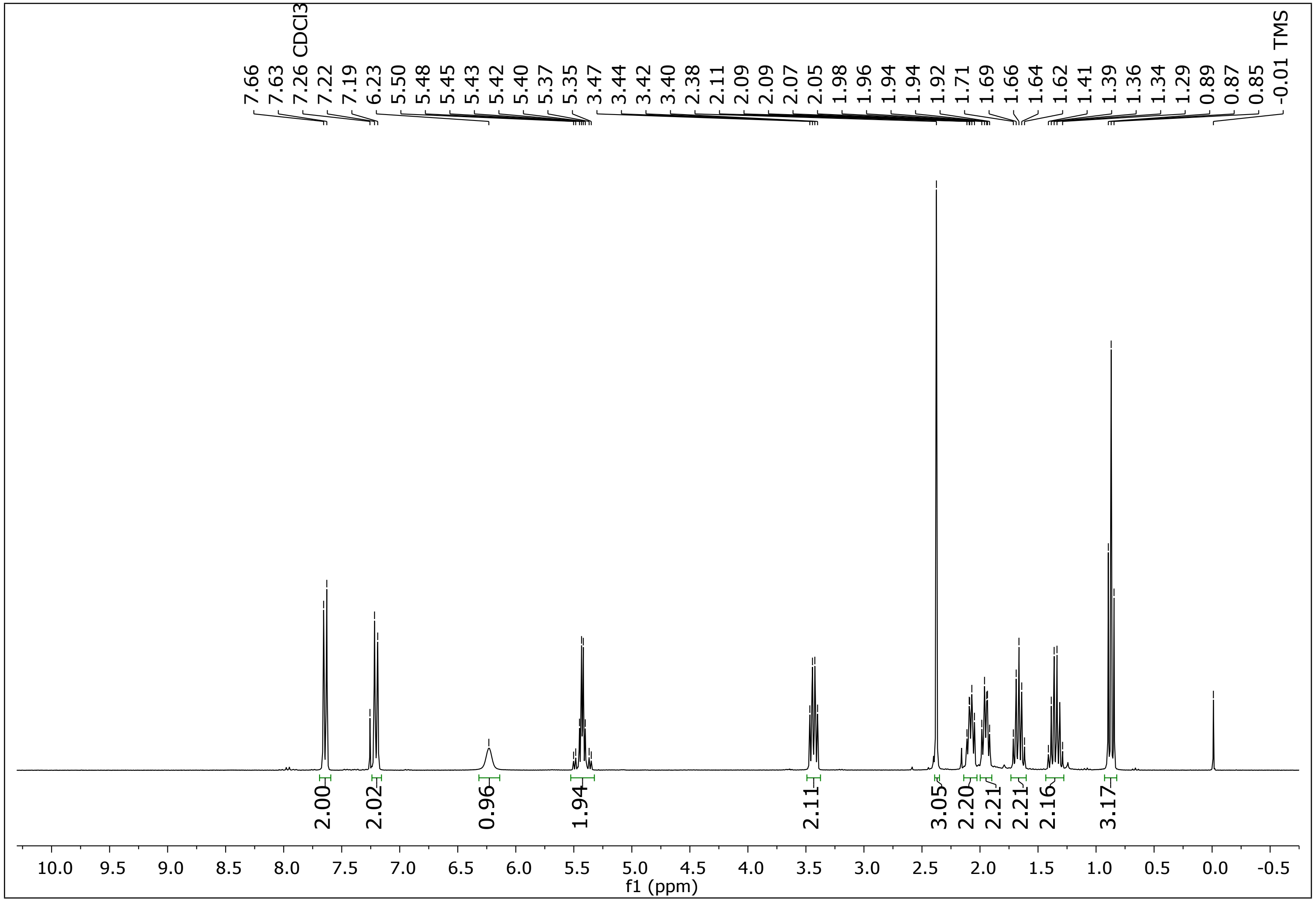

Figure S5. ^{13}C NMR spectrum (75 MHz, $CDCl_3$) of compound **2**

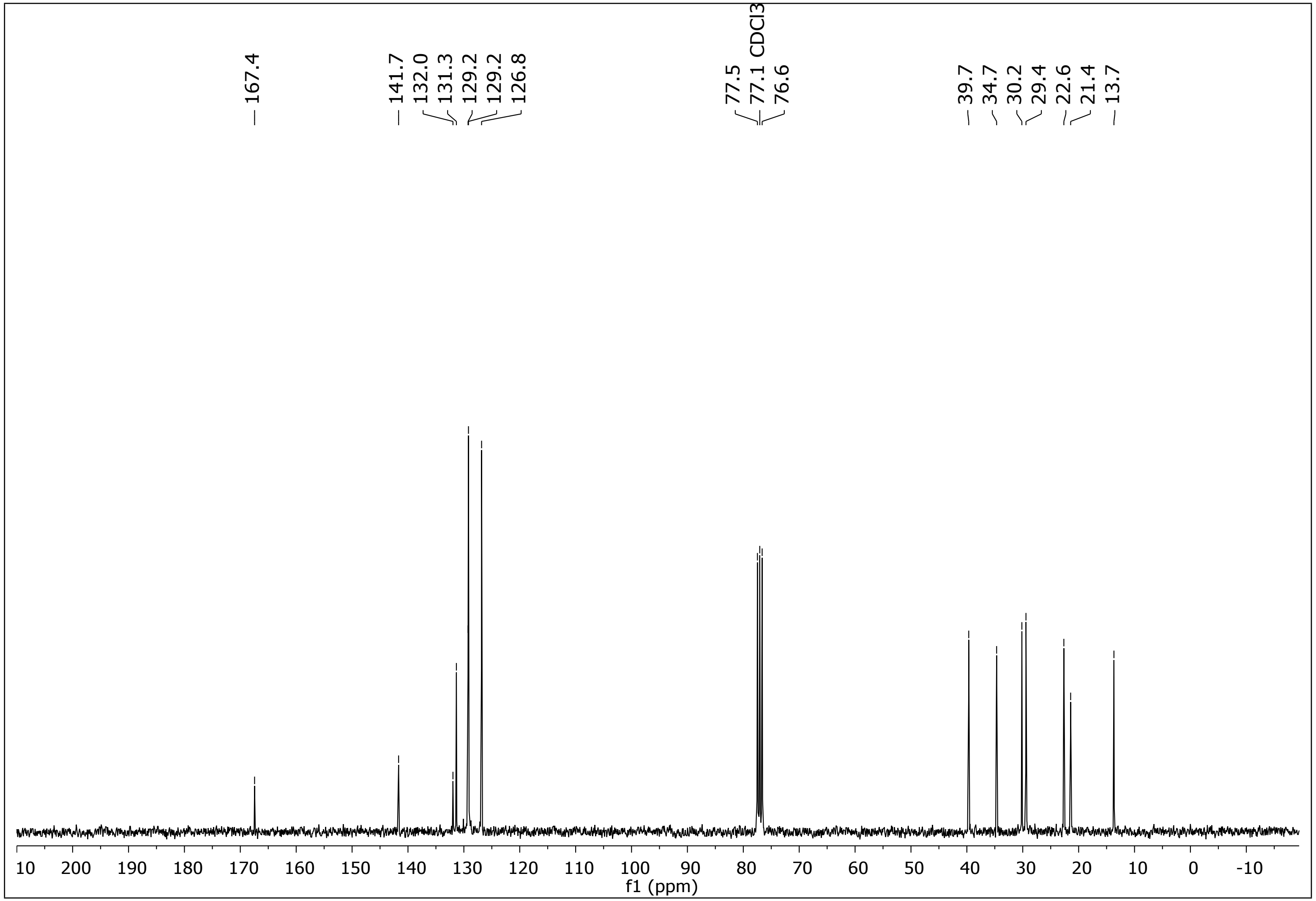

Figure S6. HRMS spectrum of compound **2**

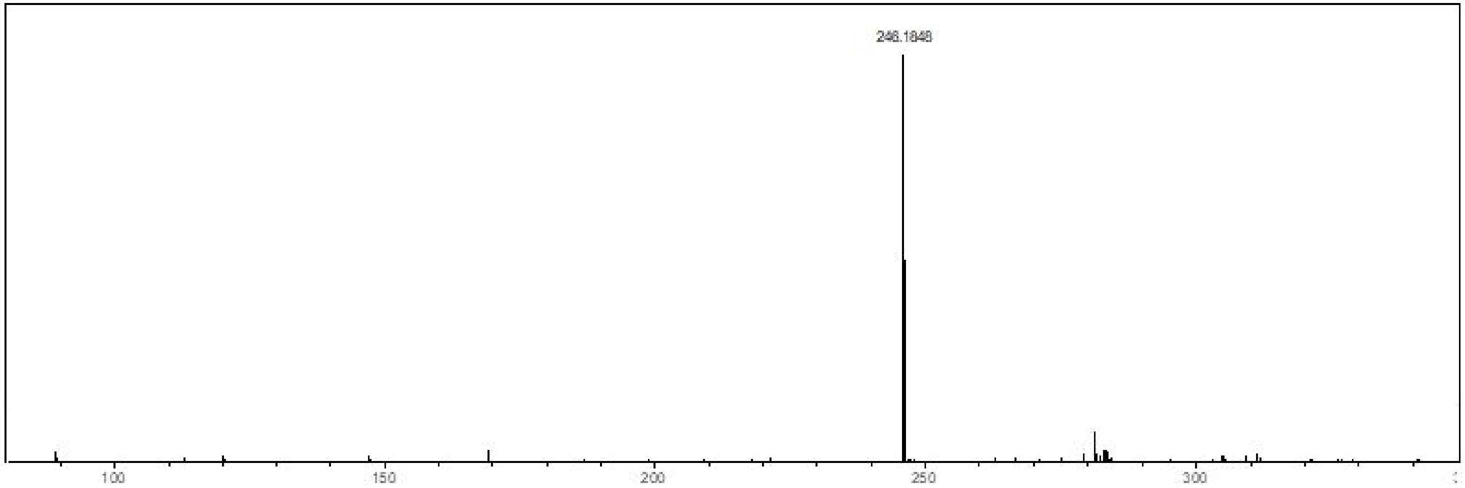

Figure S7. ¹H NMR spectrum (300 MHz, $CDCl_3$) of compound **3**

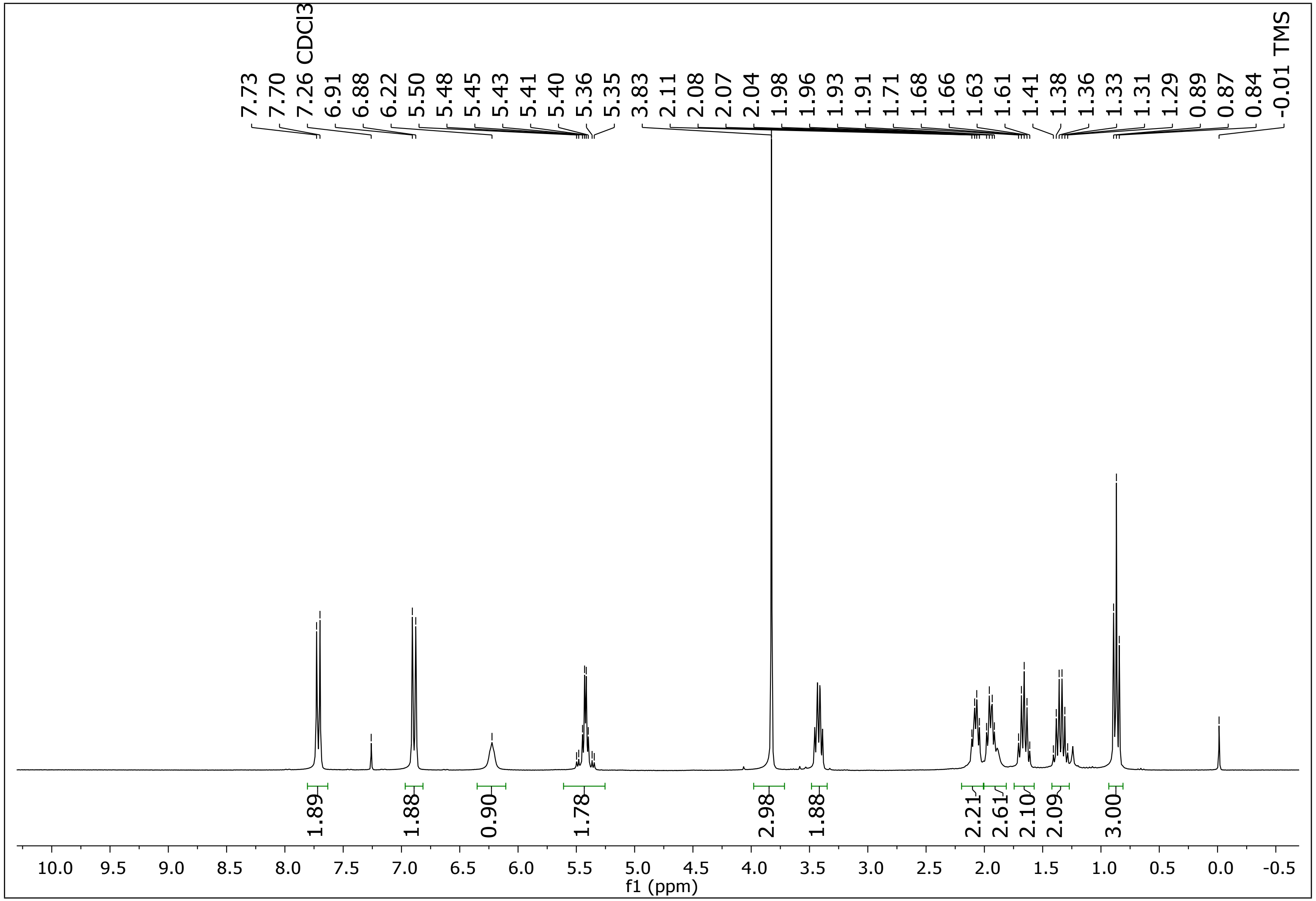

Figure S8. ^{13}C NMR spectrum (75 MHz, $CDCl_3$) of compound **3**

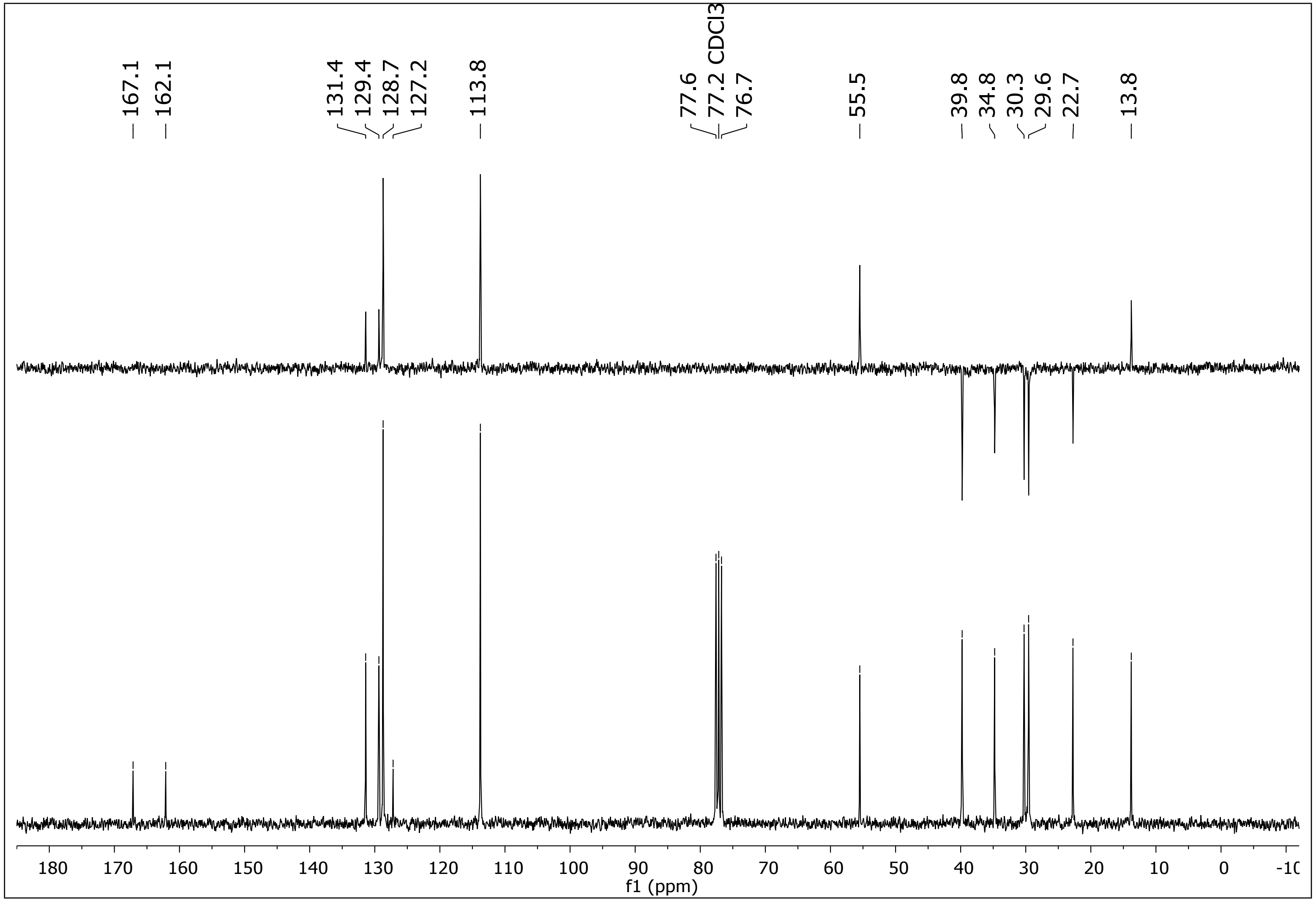

Figure S9. HRMS spectrum of compound **3**

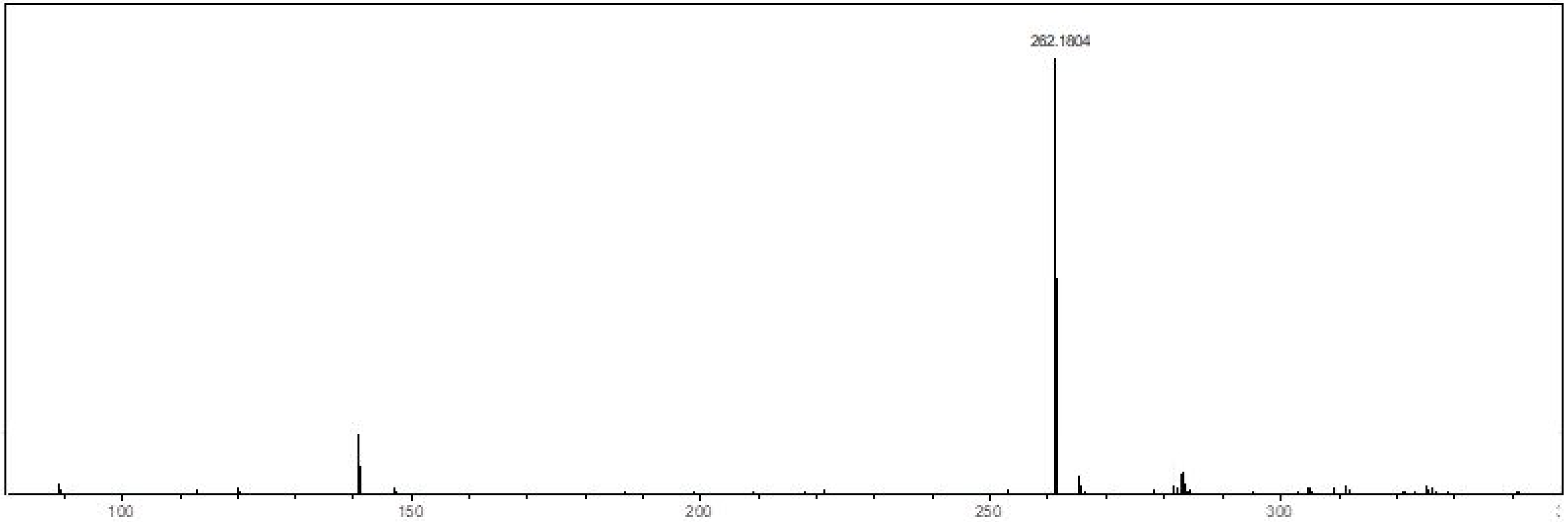

Figure S10. ^{1}H NMR spectrum (300 MHz, $CDCl_3$) of compound **4**

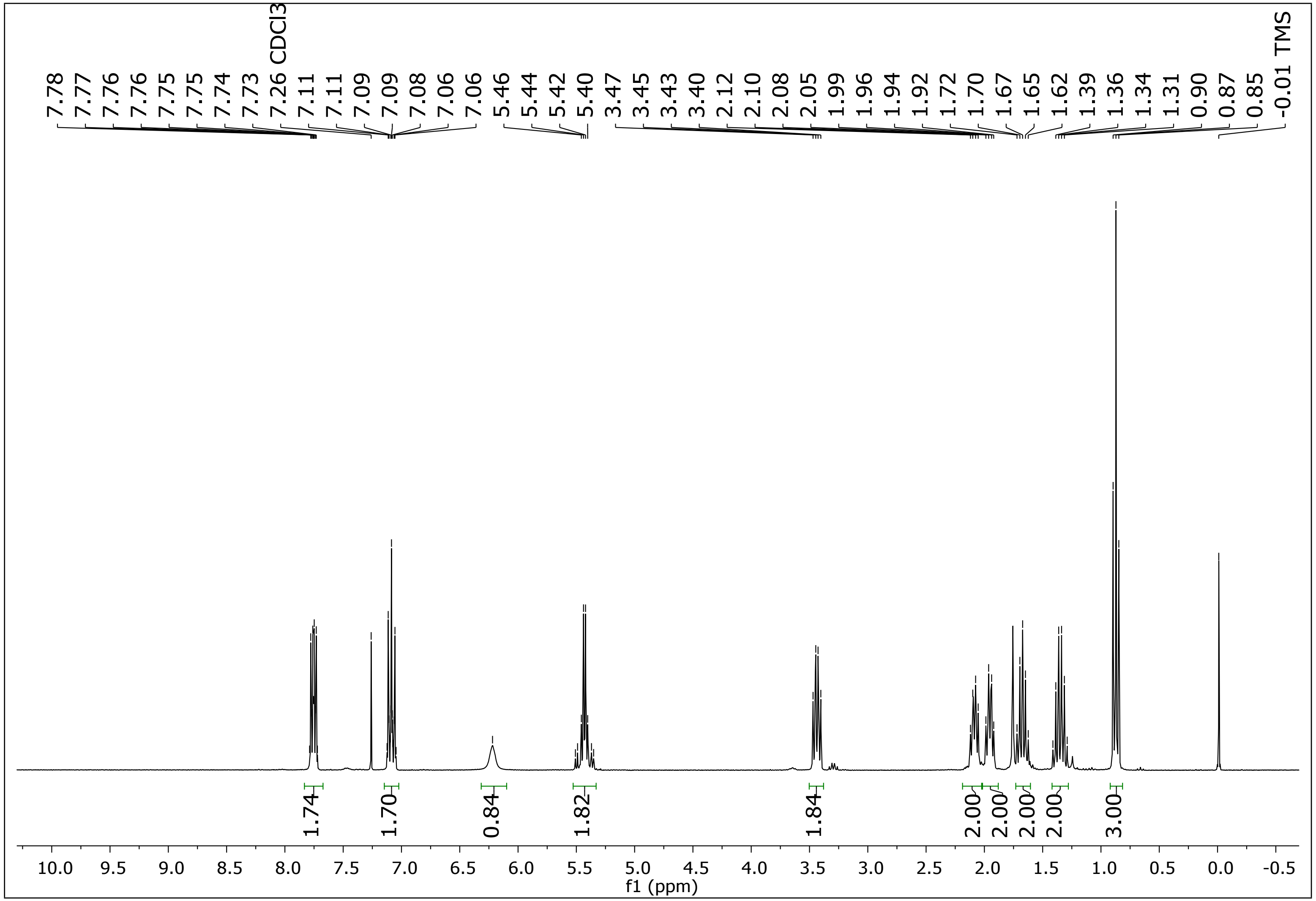

Figure S11. ^{13}C NMR spectrum (75 MHz, $CDCl_3$) of compound **4**

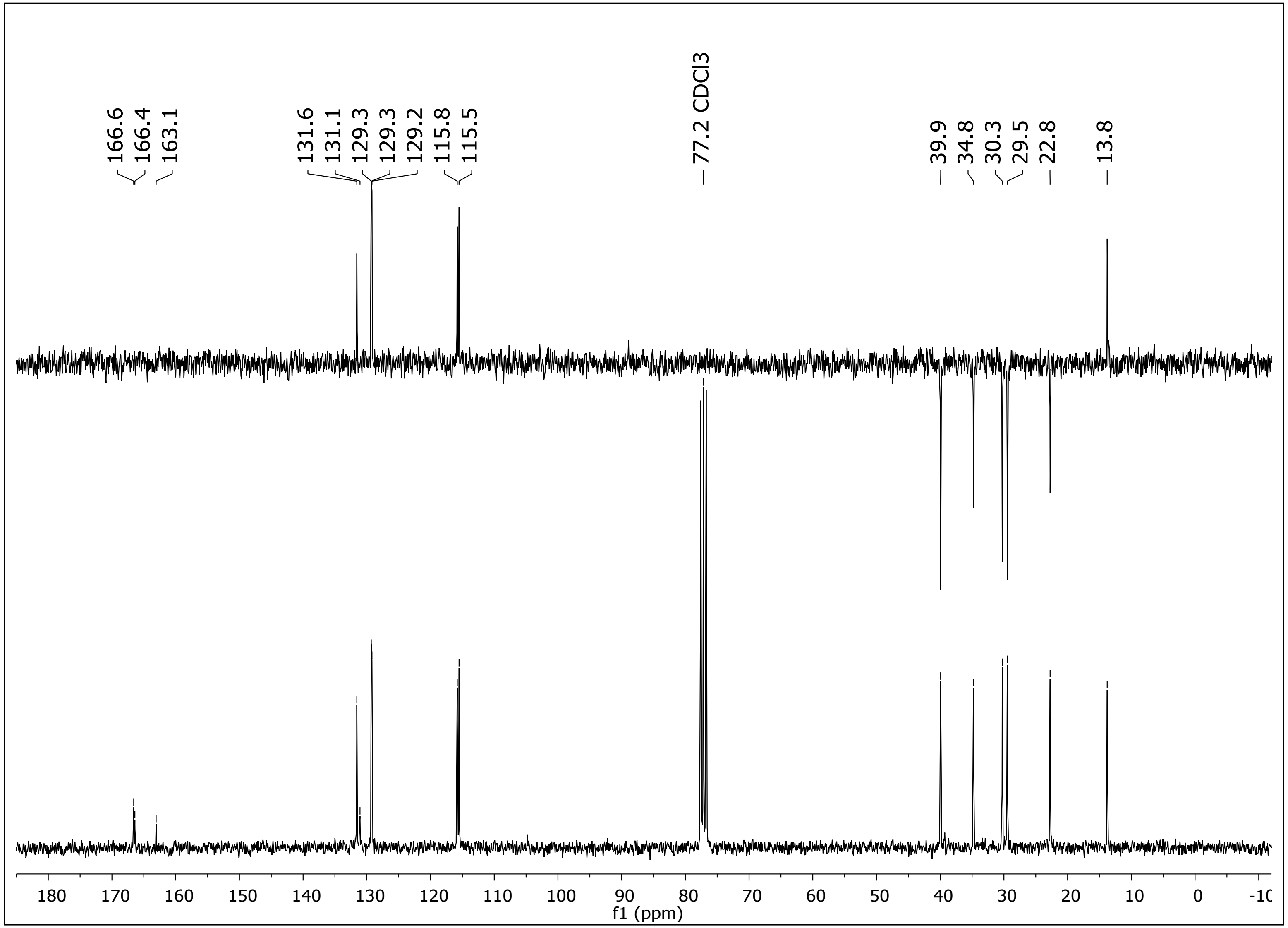

Figure S12. HRMS spectrum of compound **4**

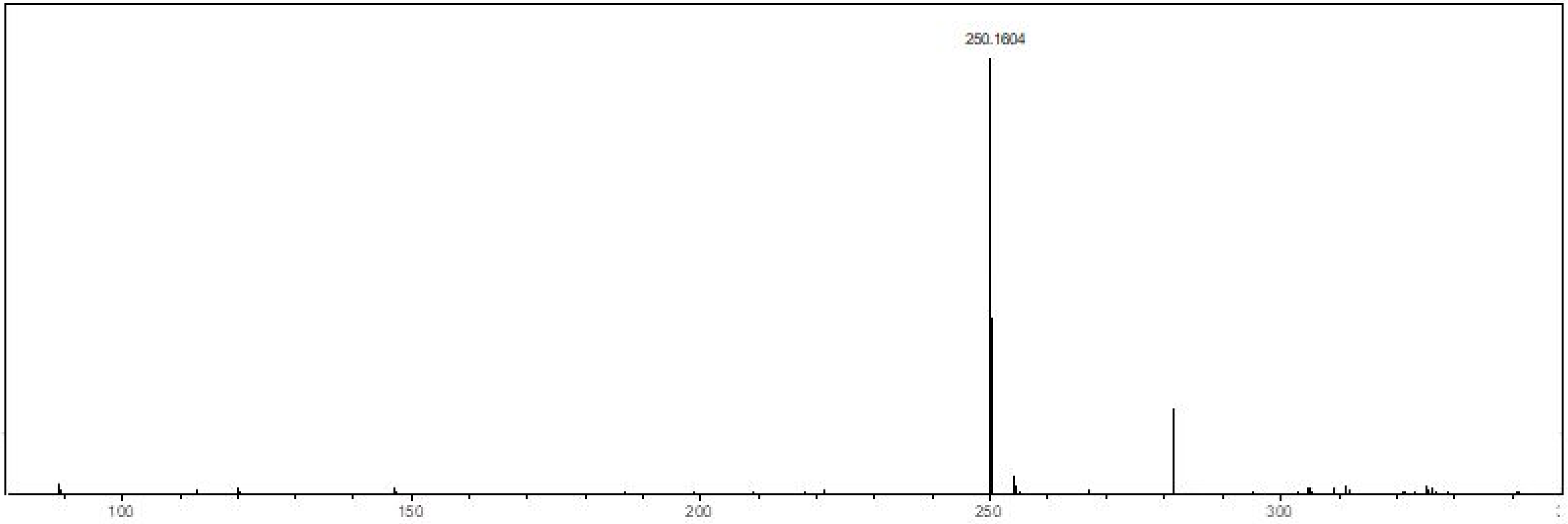

Figure S13. ^{1}H NMR spectrum (300 MHz, $CDCl_3$) of compound **5**

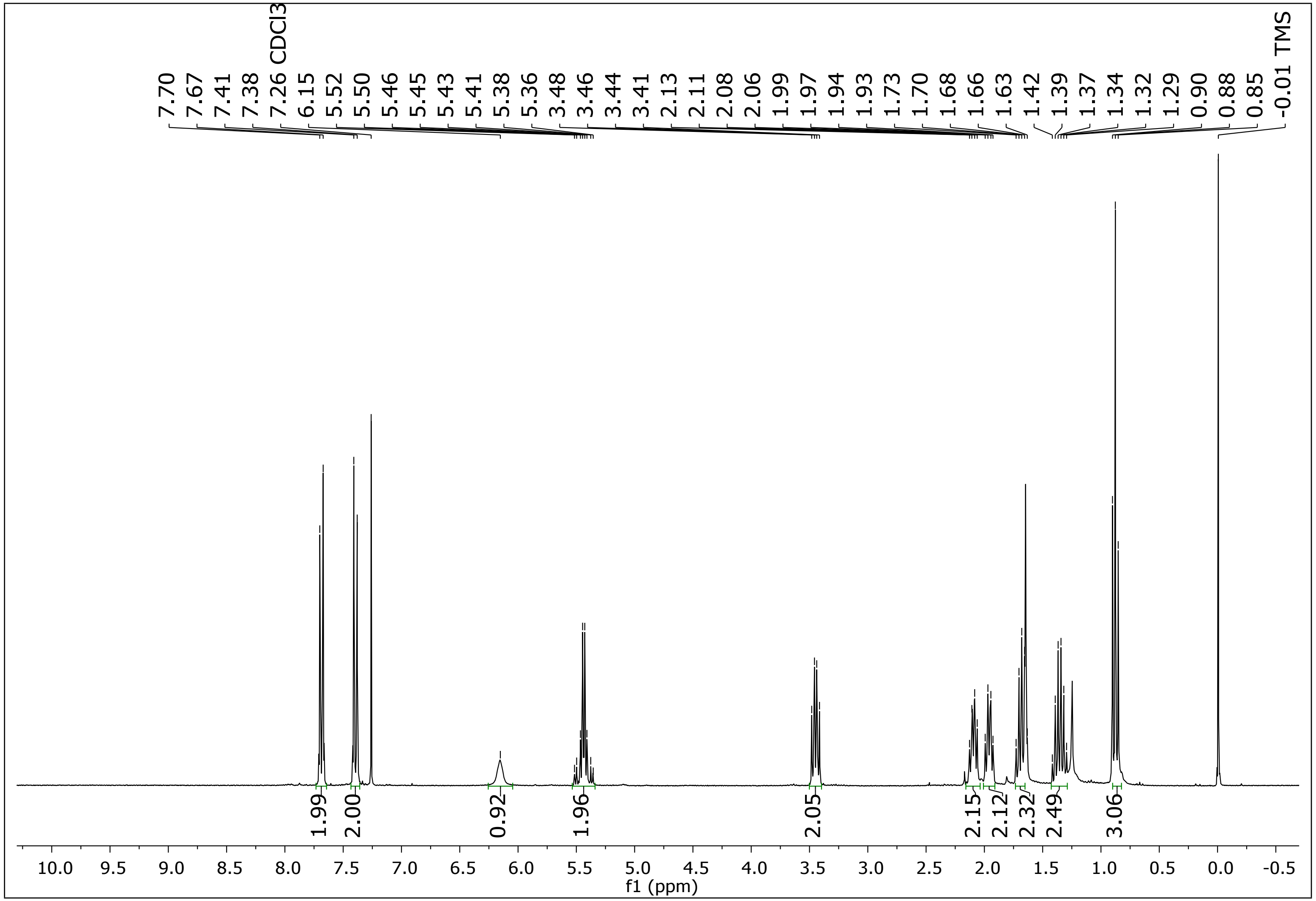

Figure S14. ^{13}C NMR spectrum (75 MHz, $CDCl_3$) of compound **5**

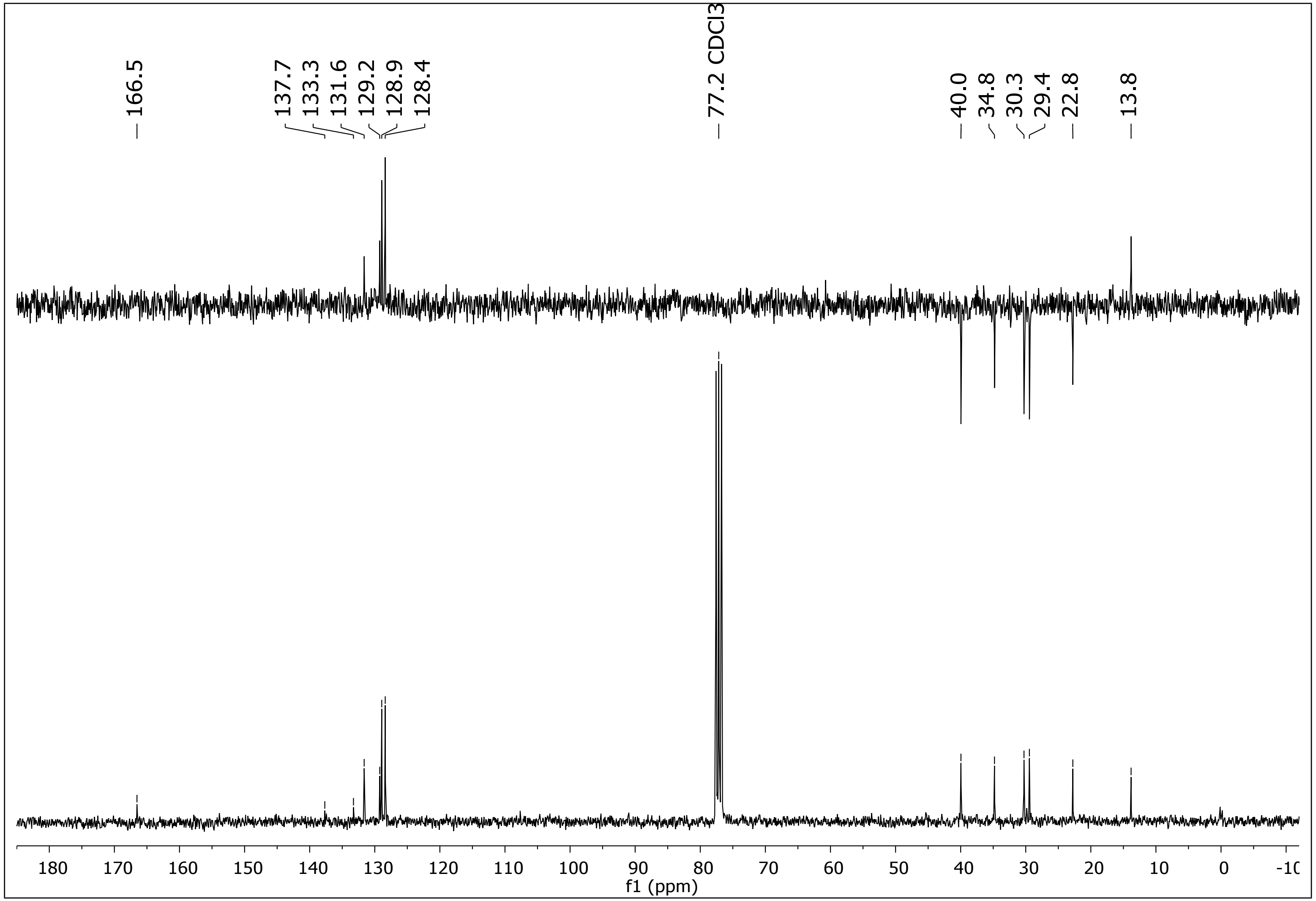

Figure S15. HRMS spectrum of compound **5**

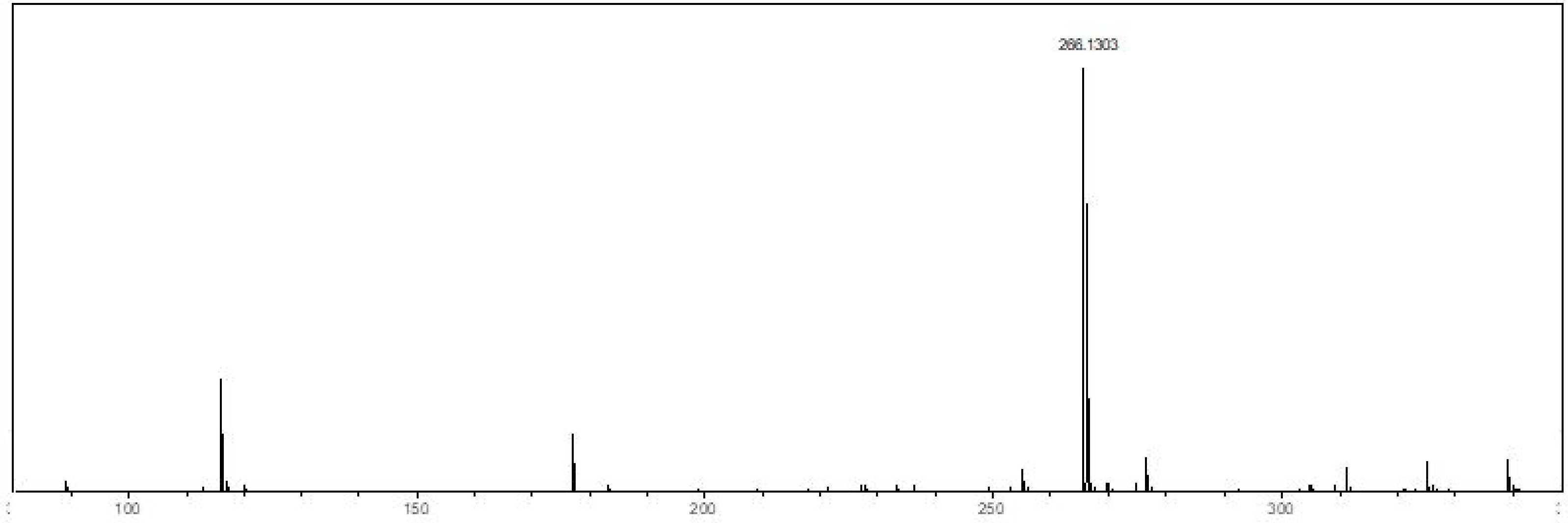

Figure S16. ^{1}H NMR spectrum (300 MHz, $CDCl_3$) of compound **6**

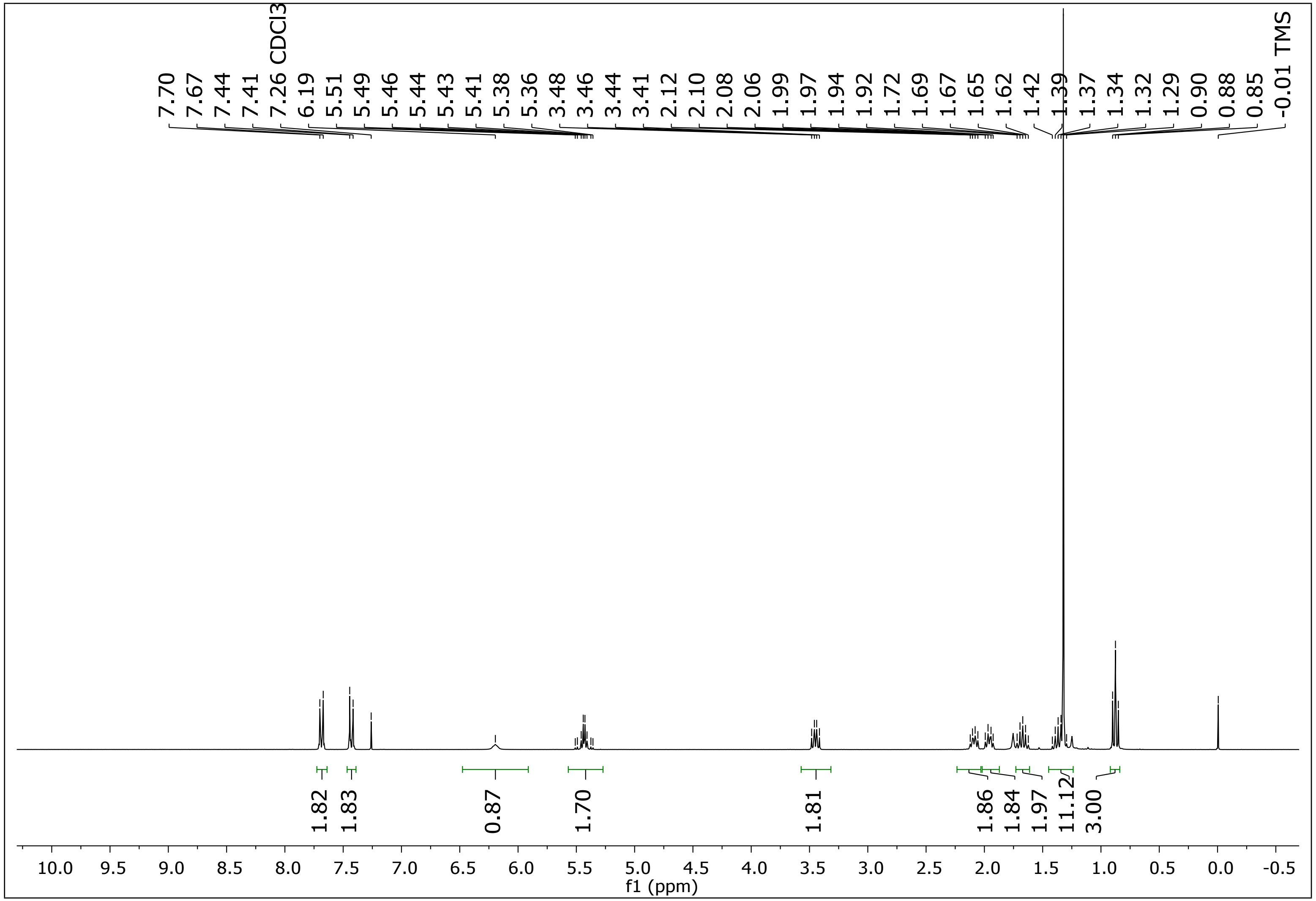

Figure S17. ^{13}C NMR spectrum (75 MHz, $CDCl_3$) of compound **6**

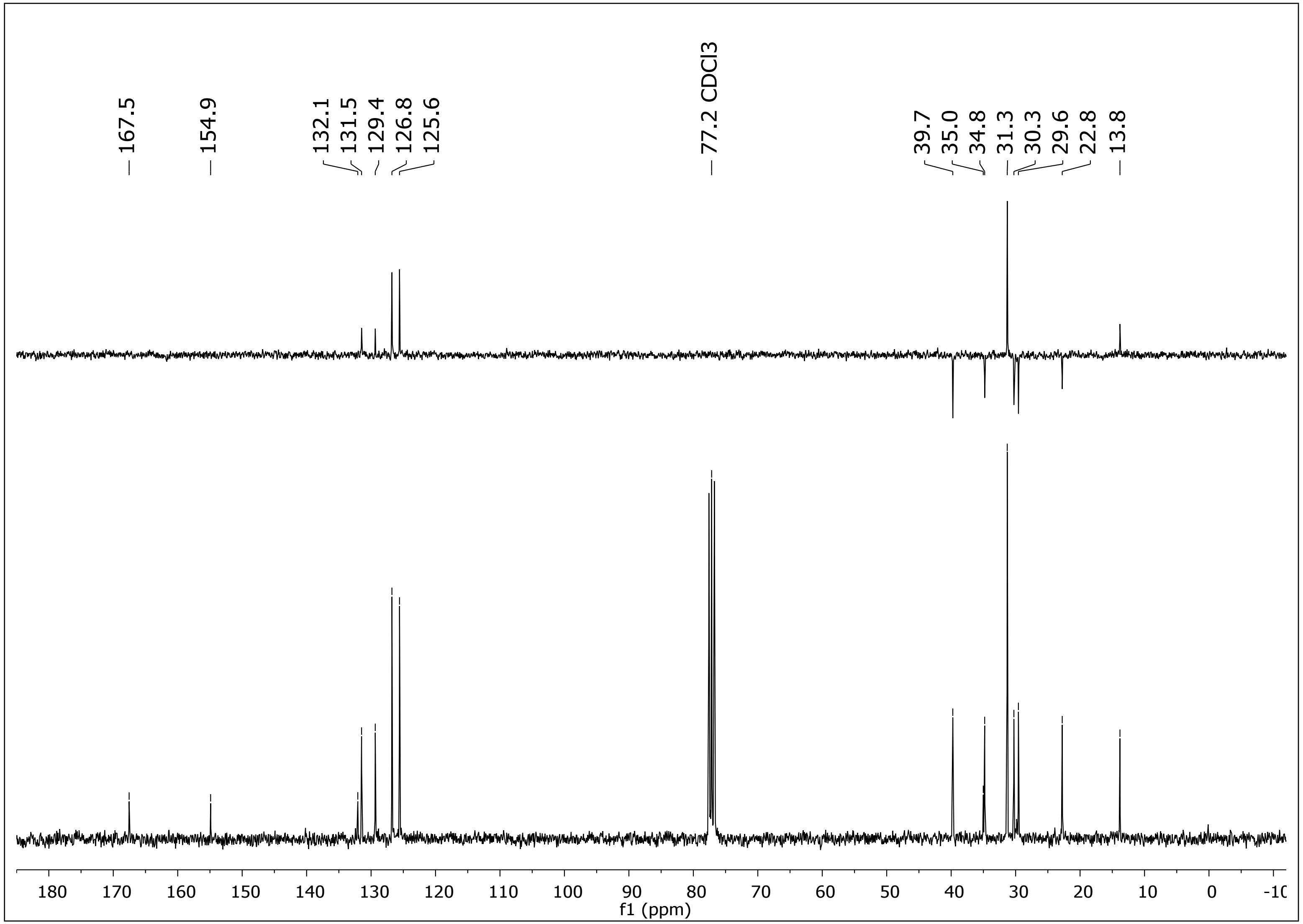

Figure S18. HRMS spectrum of compound **6**

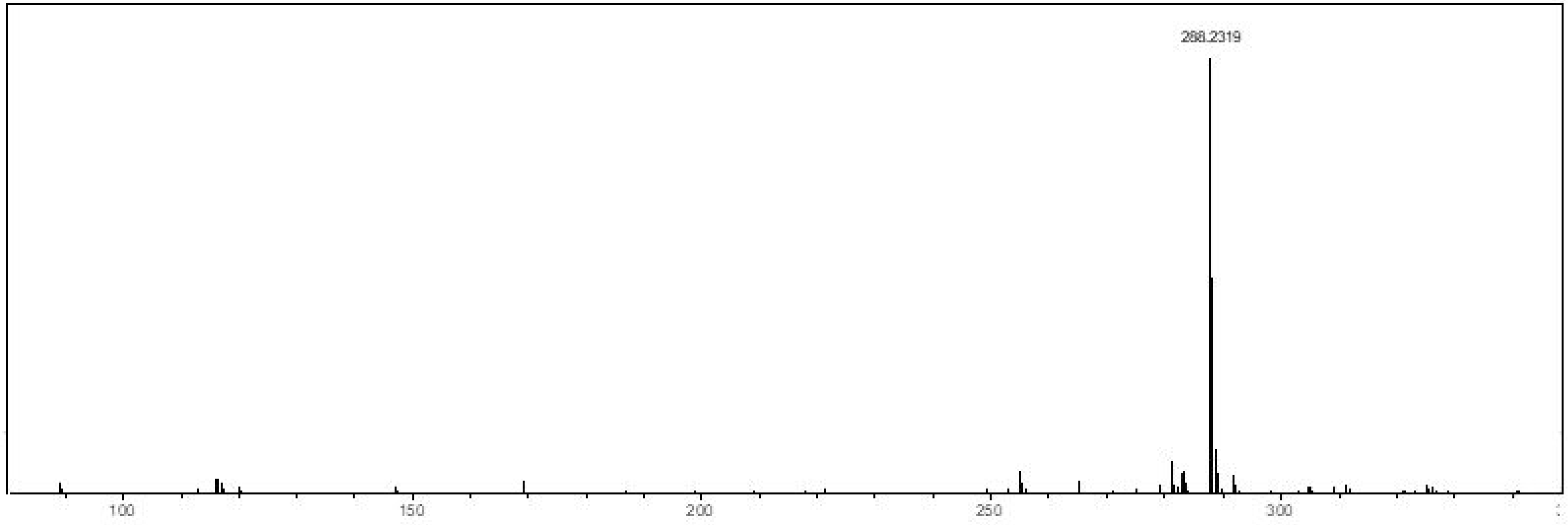

Figure S19. ^{1}H NMR spectrum (300 MHz, $CDCl_3$) of compound **7**

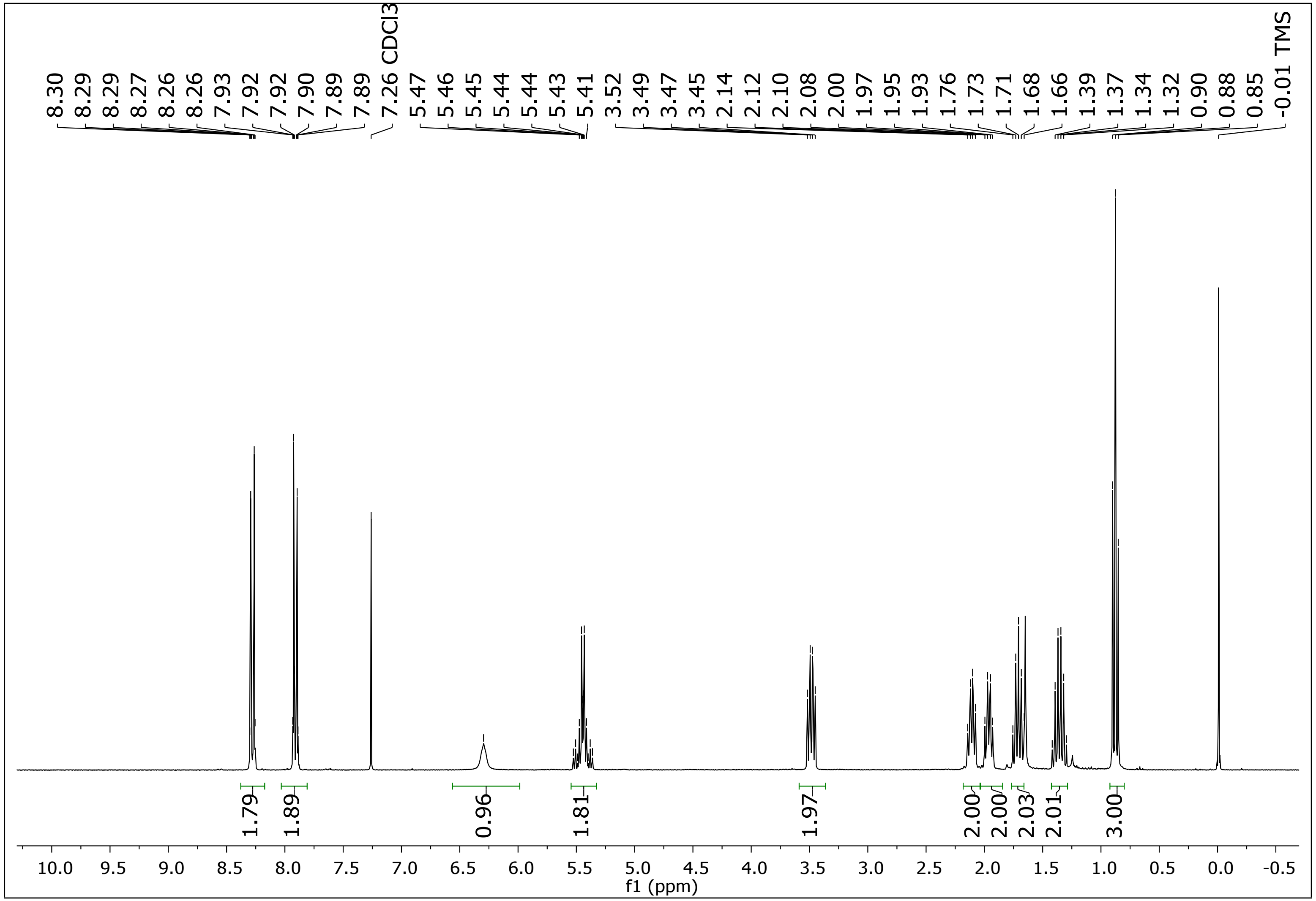

Figure S20. ^{13}C NMR spectrum (75 MHz, $CDCl_3$) of compound **7**

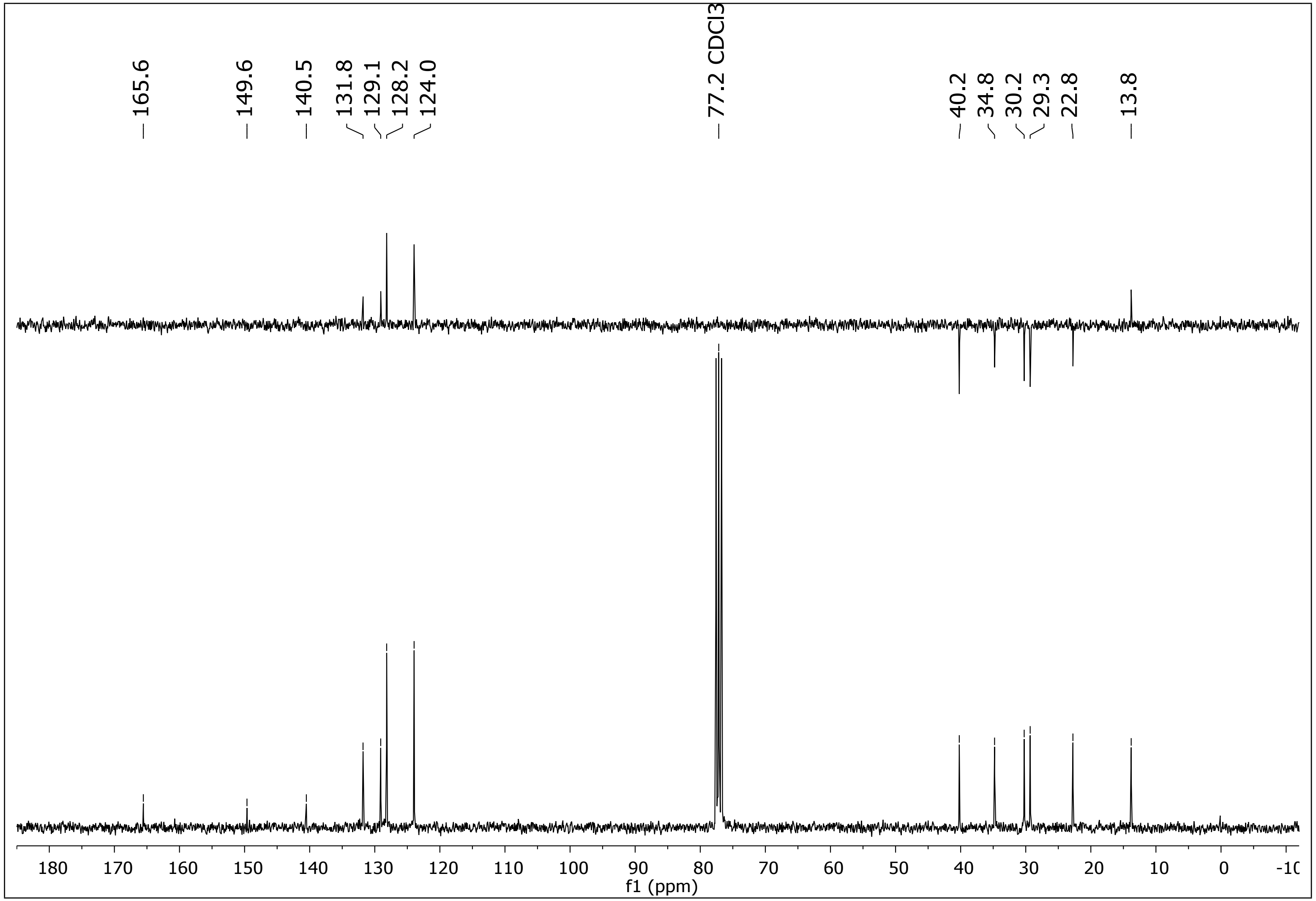

Figure S21. HRMS spectrum of compound **7**

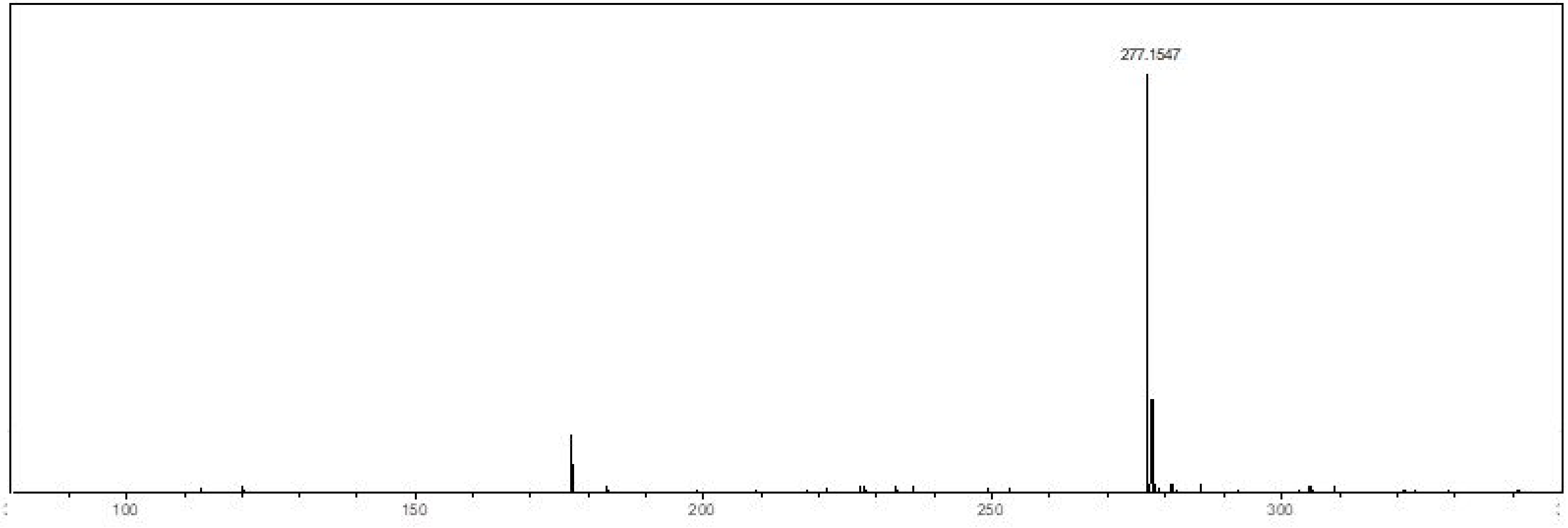

Figure S22. ^{1}H NMR spectrum (300 MHz, $CDCl_3$) of compound **8**

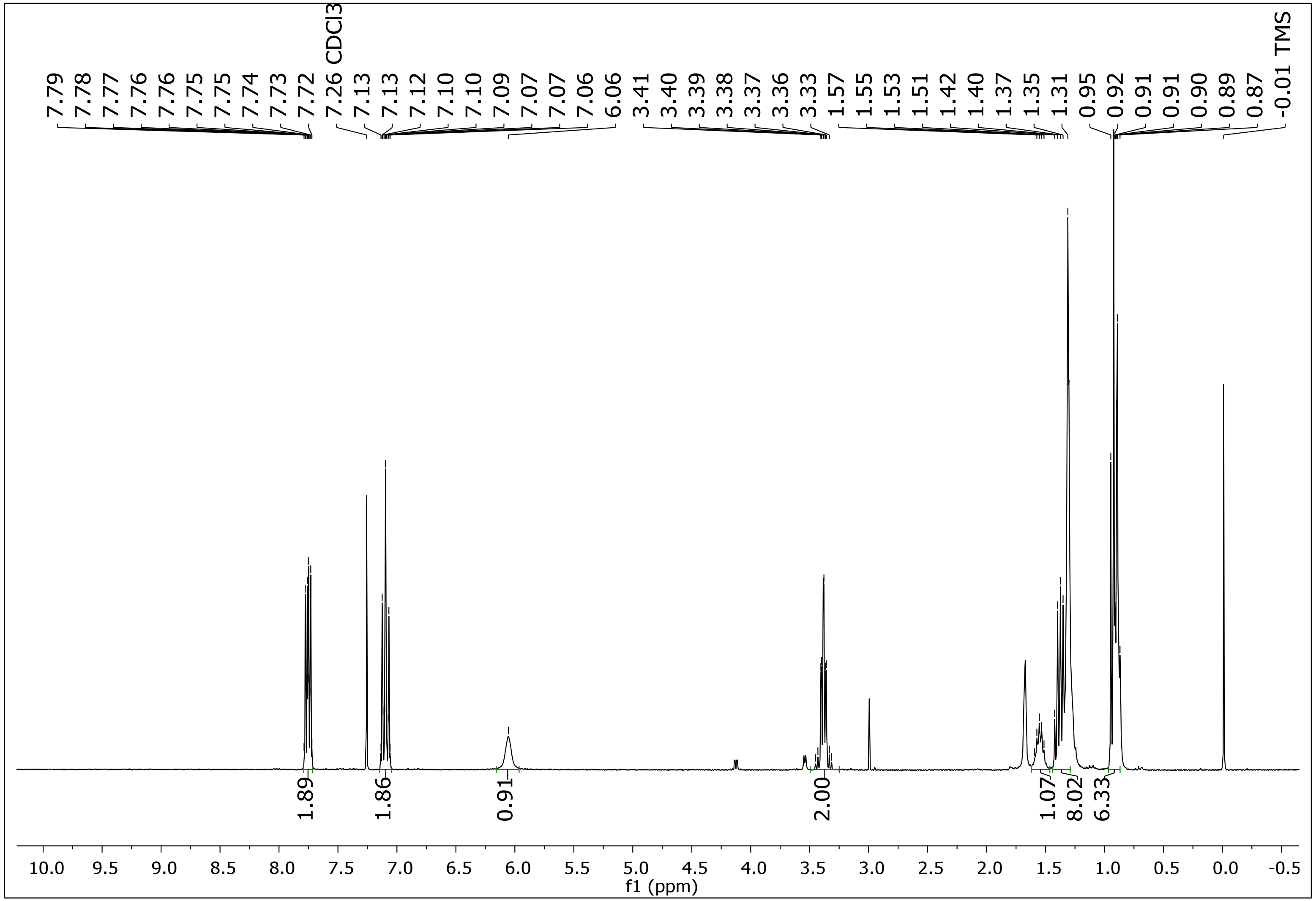

Figure S23. ^{13}C NMR spectrum (75 MHz, $CDCl_3$) of compound **8**

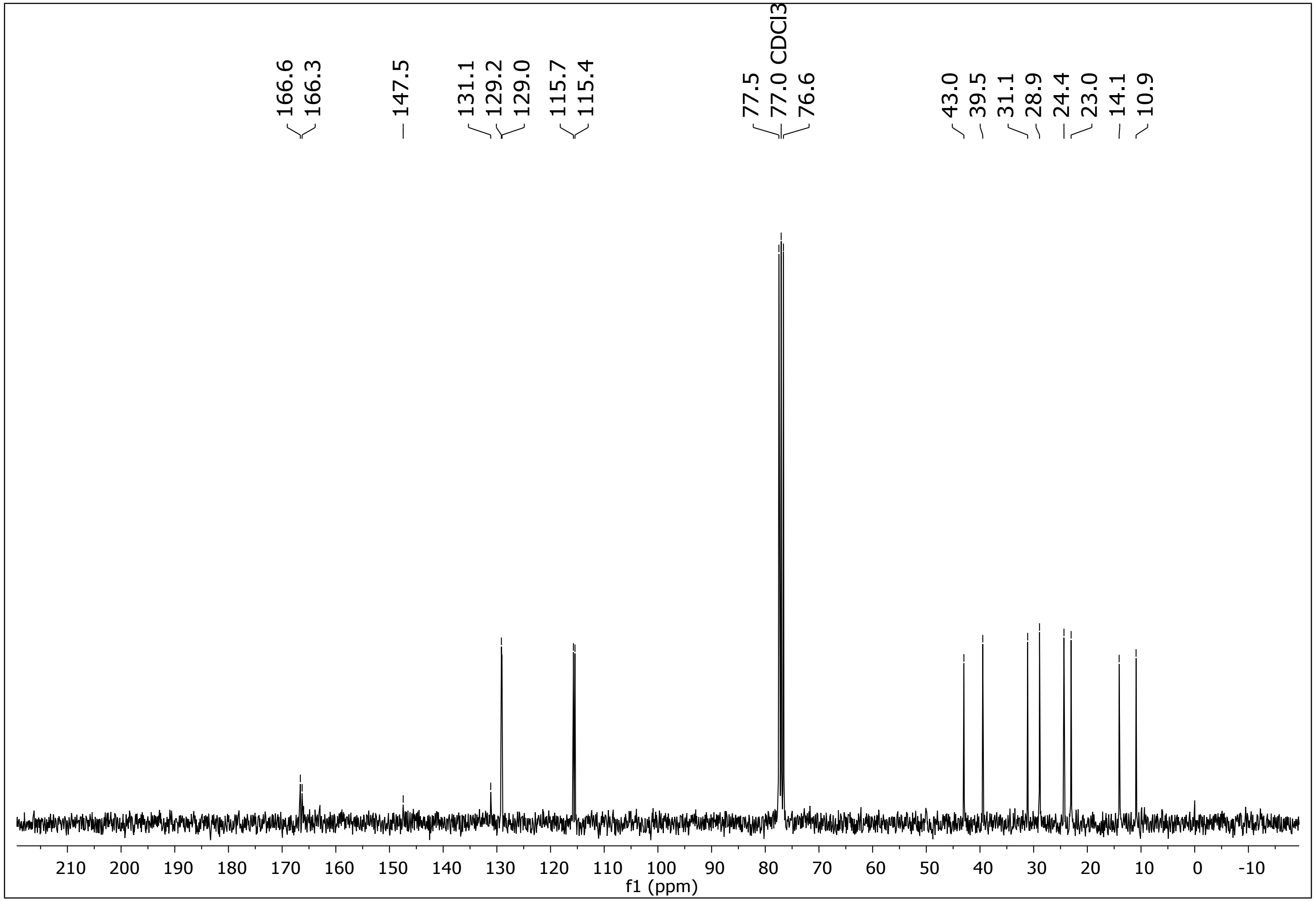

Figure S24. HRMS spectrum of compound **8**

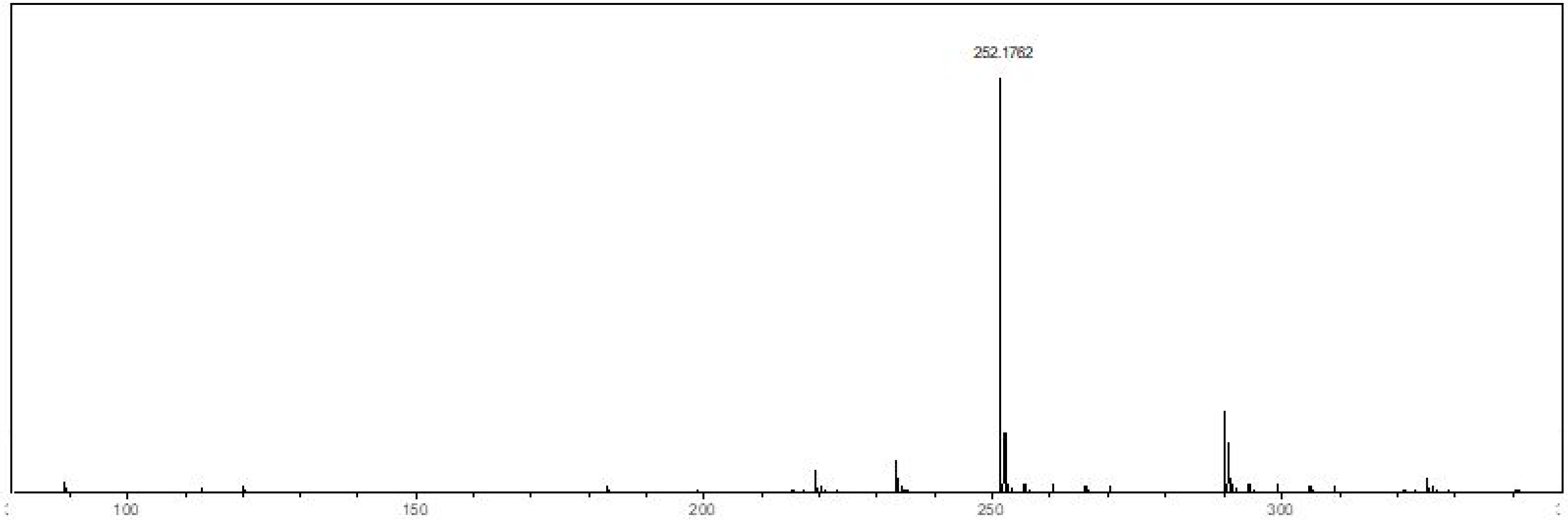

Figure S25. ^{1}H NMR spectrum (300 MHz, $CDCl_3$) of compound **9**

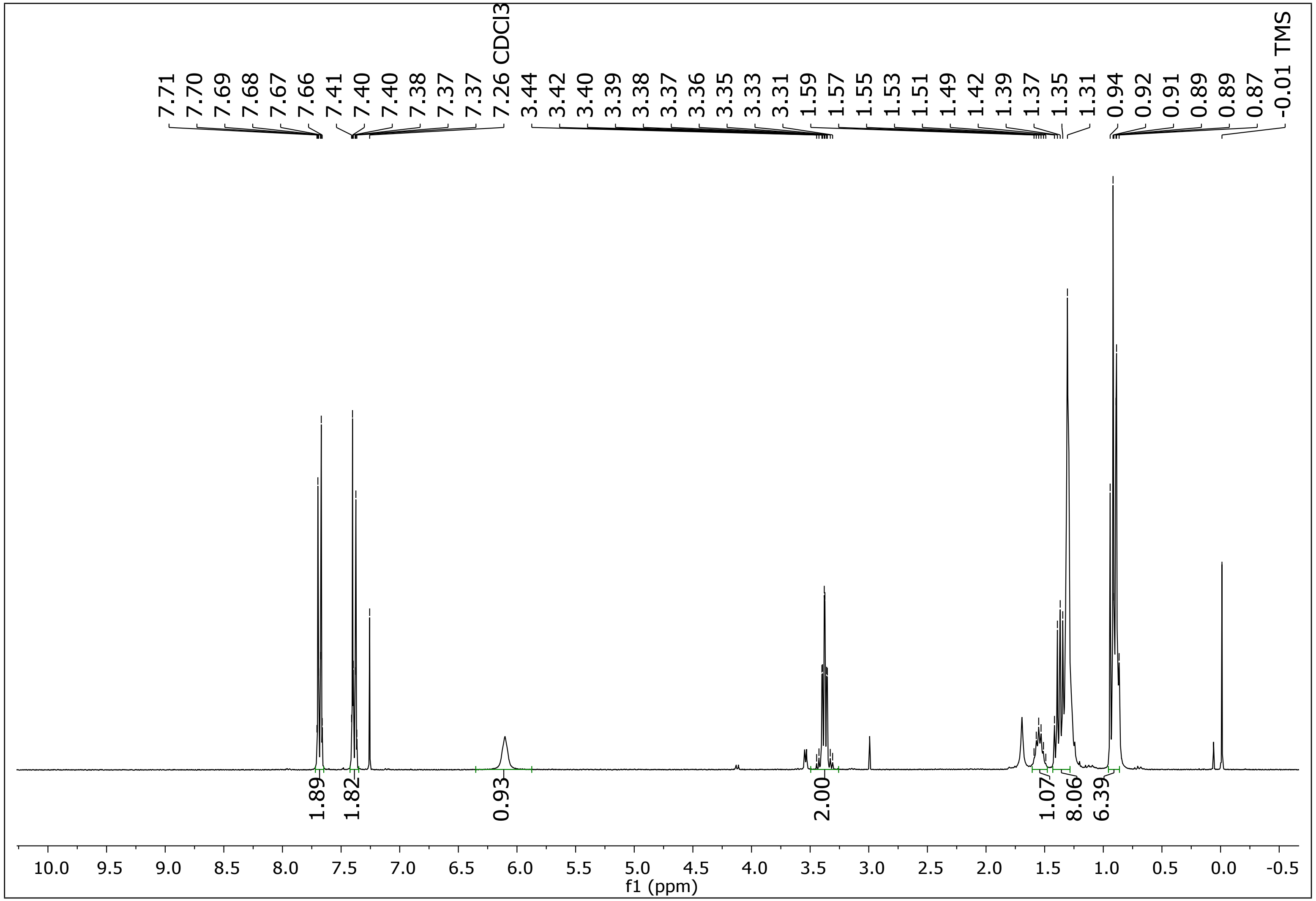

Figure S26. ^{13}C NMR spectrum (75 MHz, $CDCl_3$) of compound **9**

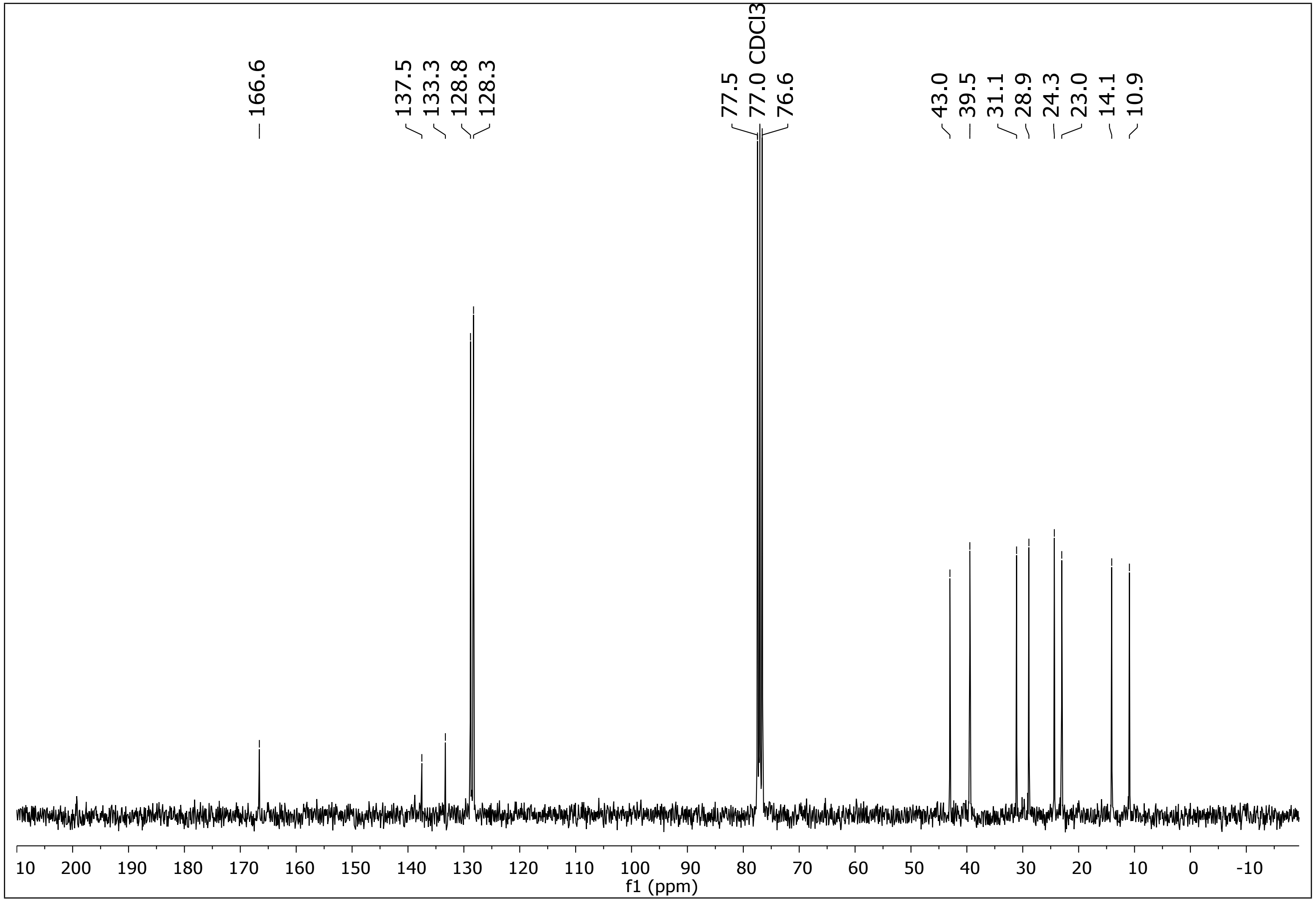

Figure S27. HRMS spectrum of compound **9**

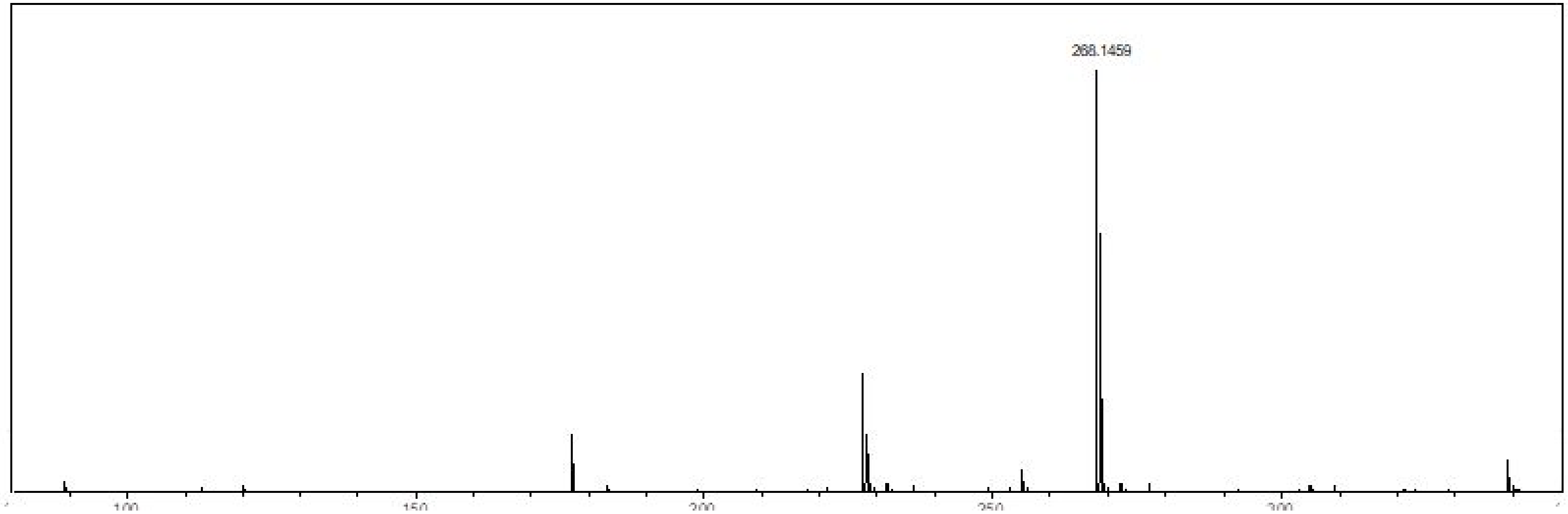

Figure S28. ^{1}H NMR spectrum (300 MHz, $CDCl_3$) of compound **10**

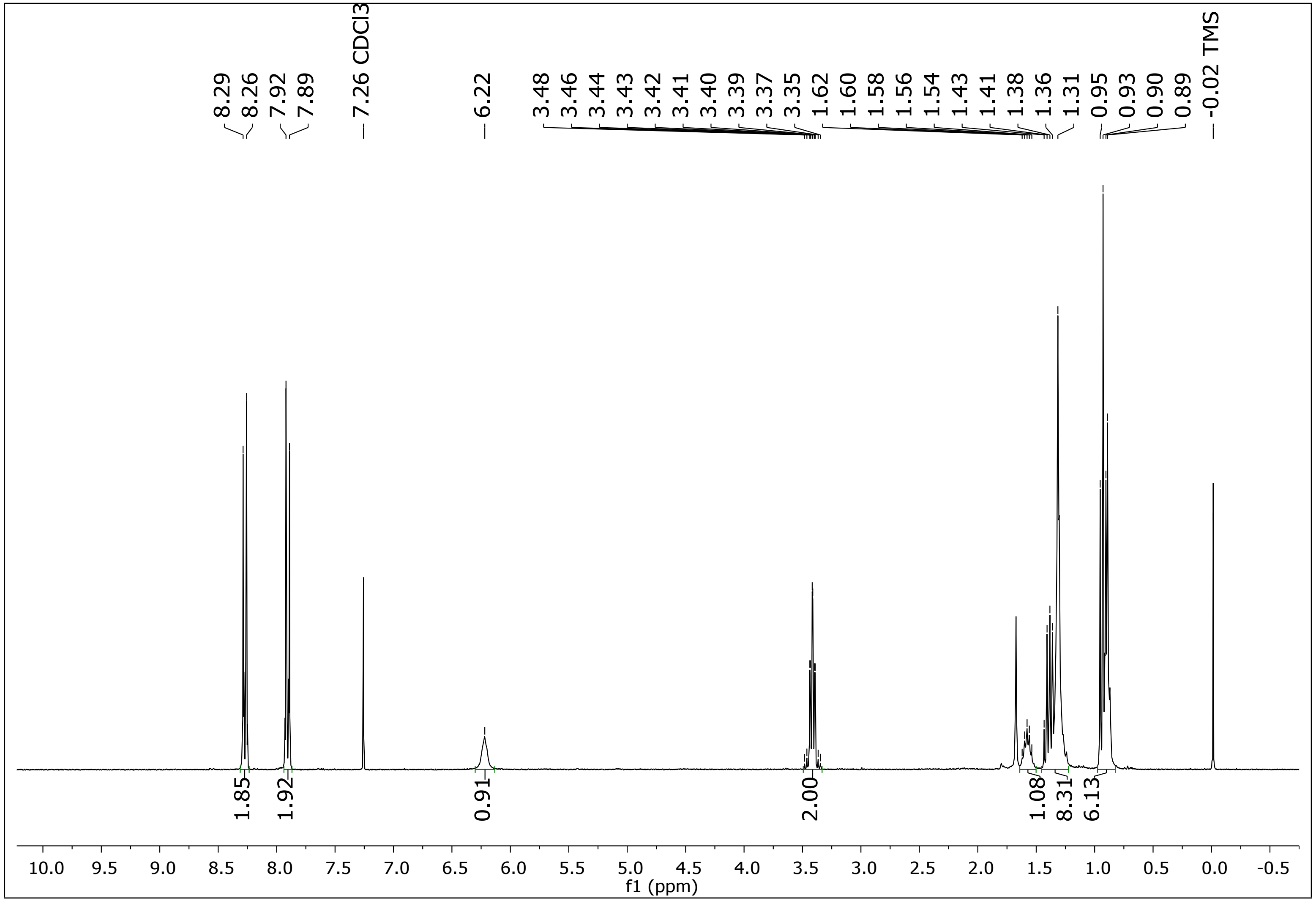

Figure S29. ^{13}C NMR spectrum (75 MHz, $CDCl_3$) of compound **10**

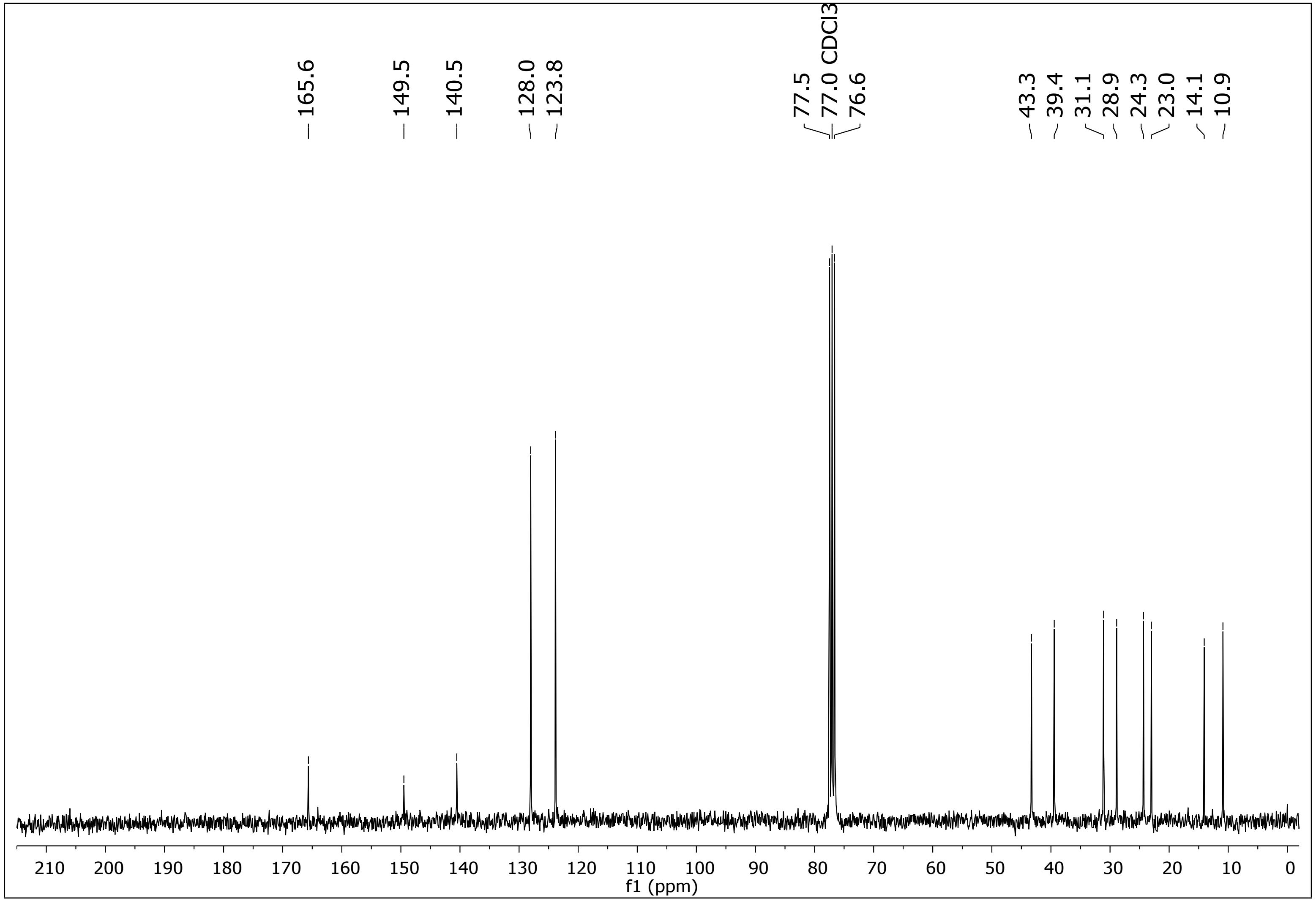

Figure S30. HRMS spectrum of compound **10**

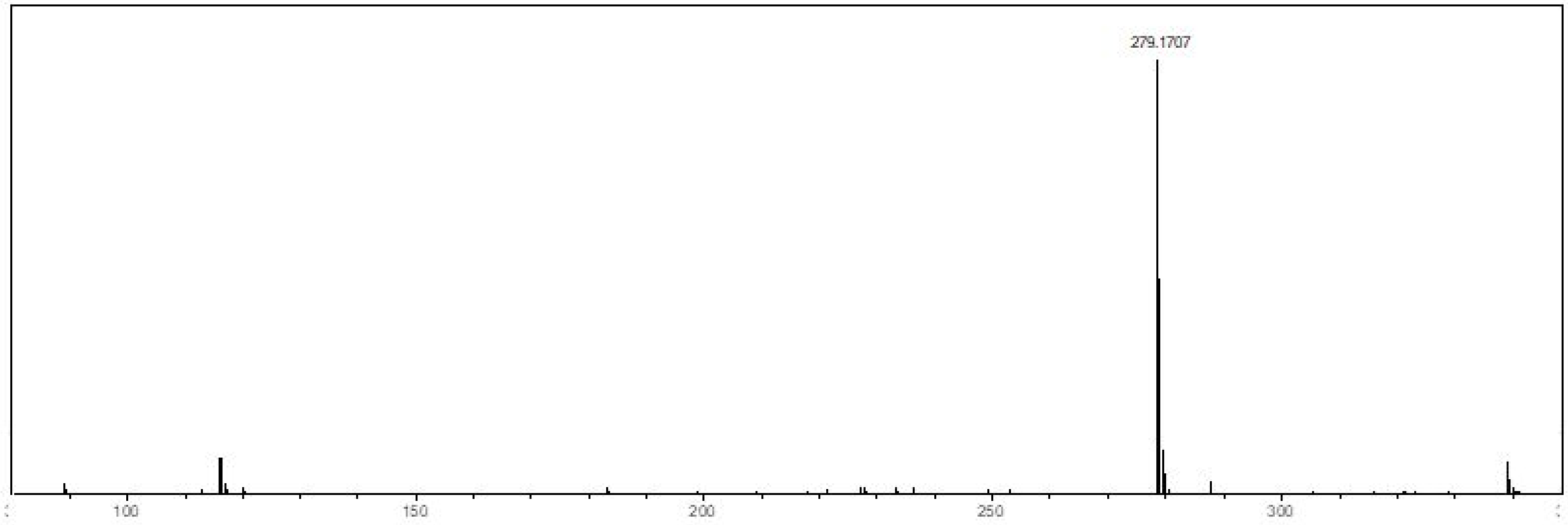

Figure S31. ^{1}H NMR spectrum (300 MHz, $CDCl_3$) of compound **11**

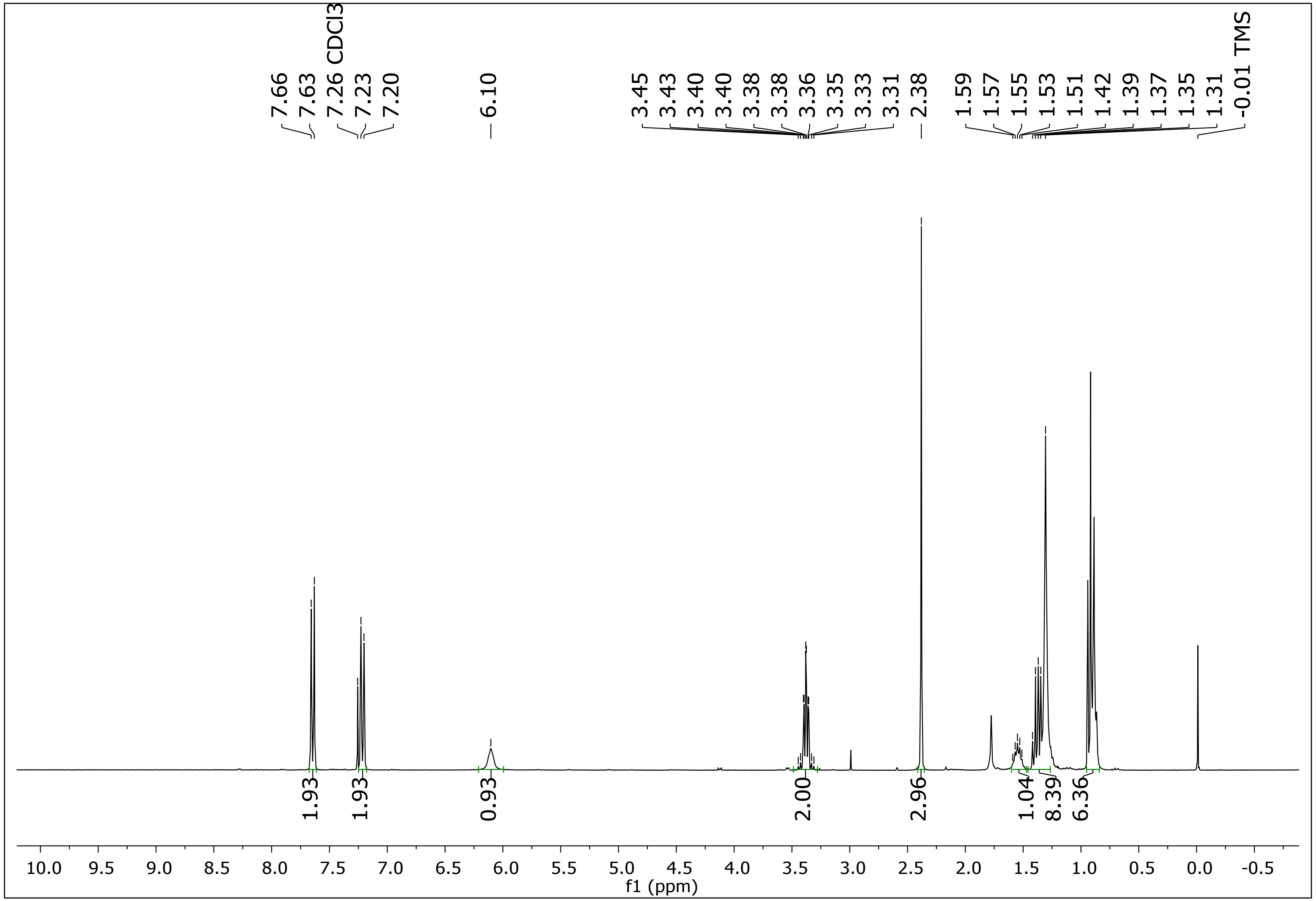

Figure S32. ^{13}C NMR spectrum (75 MHz, $CDCl_3$) of compound **11**

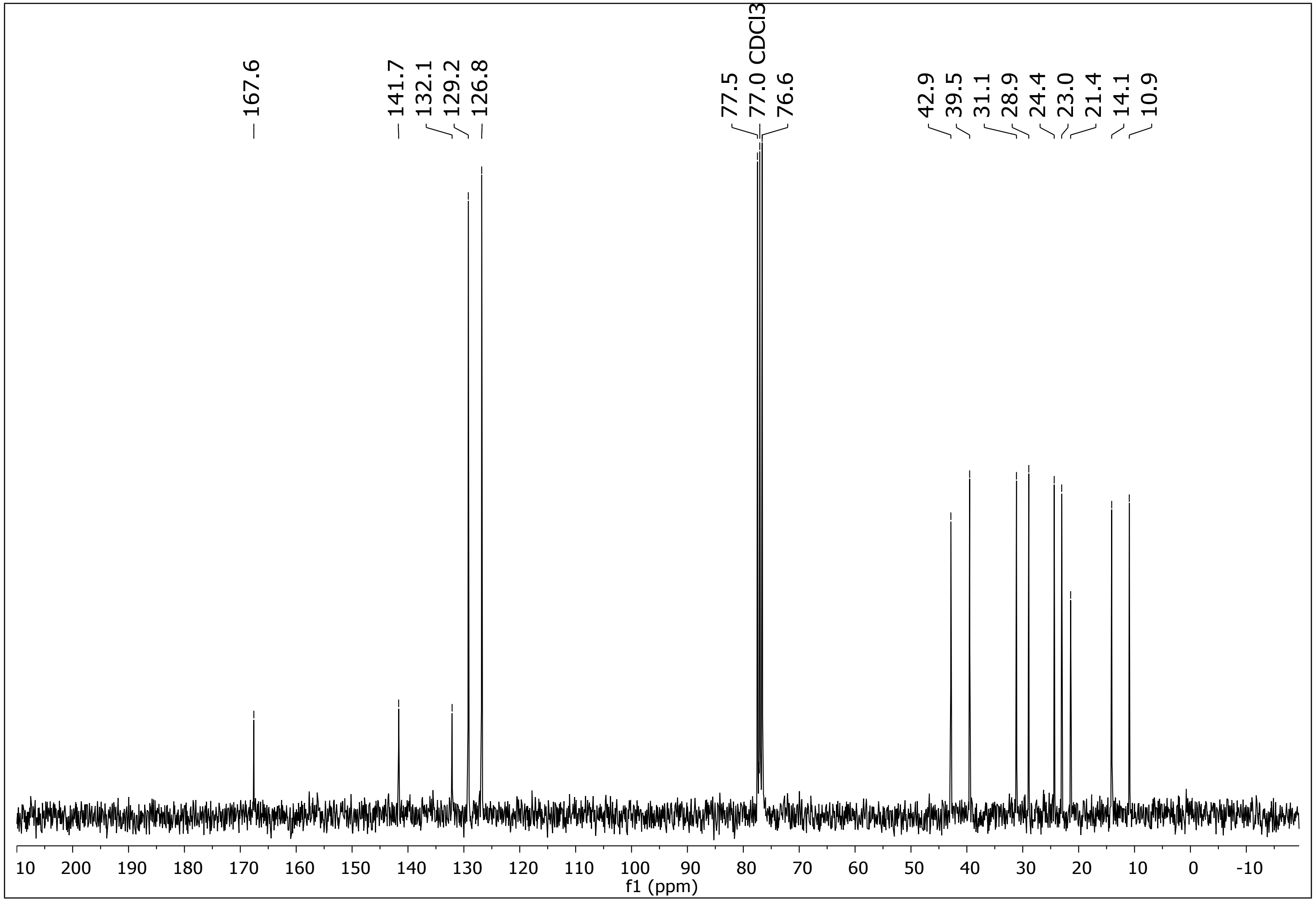

Figure S33. HRMS spectrum of compound **11**

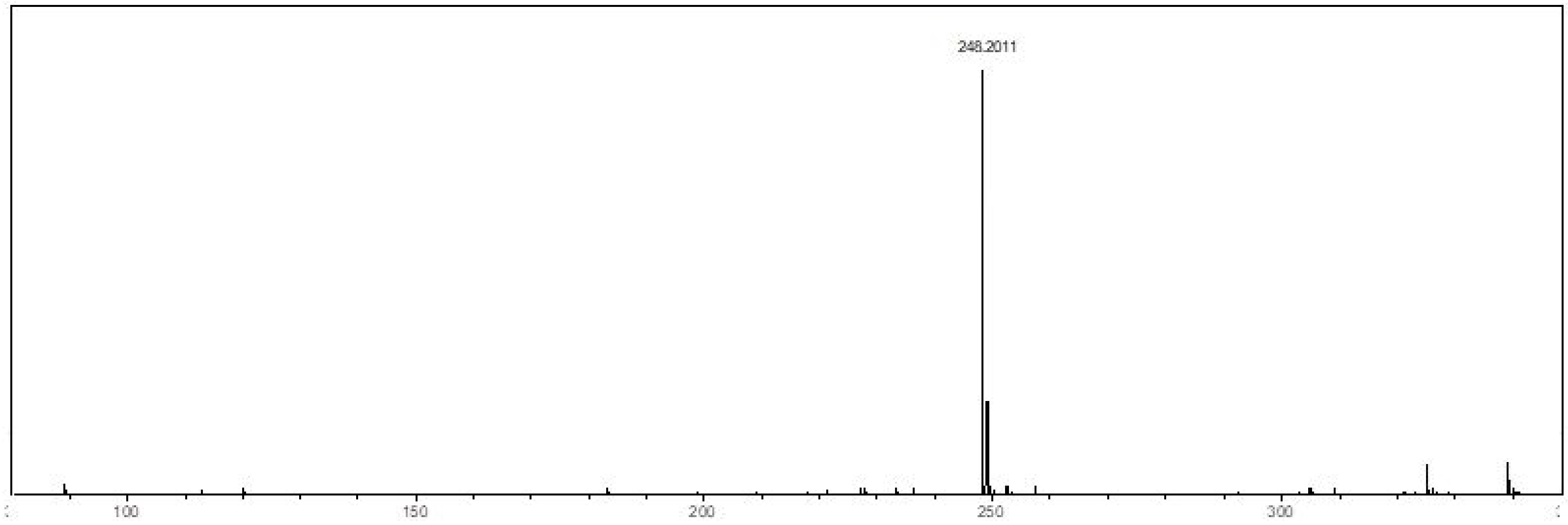

Figure S34. ^{1}H NMR spectrum (300 MHz, $CDCl_3$) of compound **12**

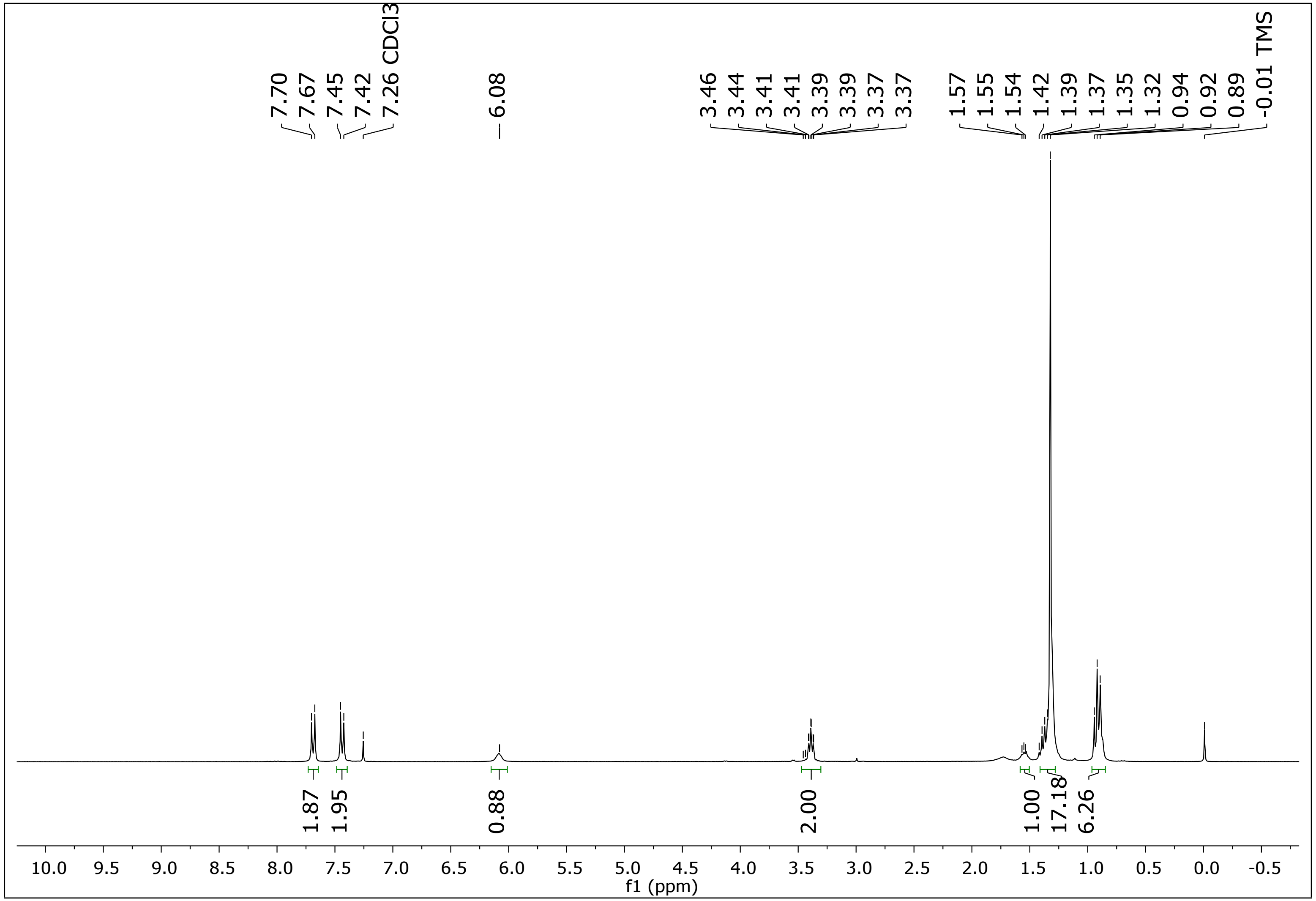

Figure S35. ^{13}C NMR spectrum (75 MHz, $CDCl_3$) of compound **12**

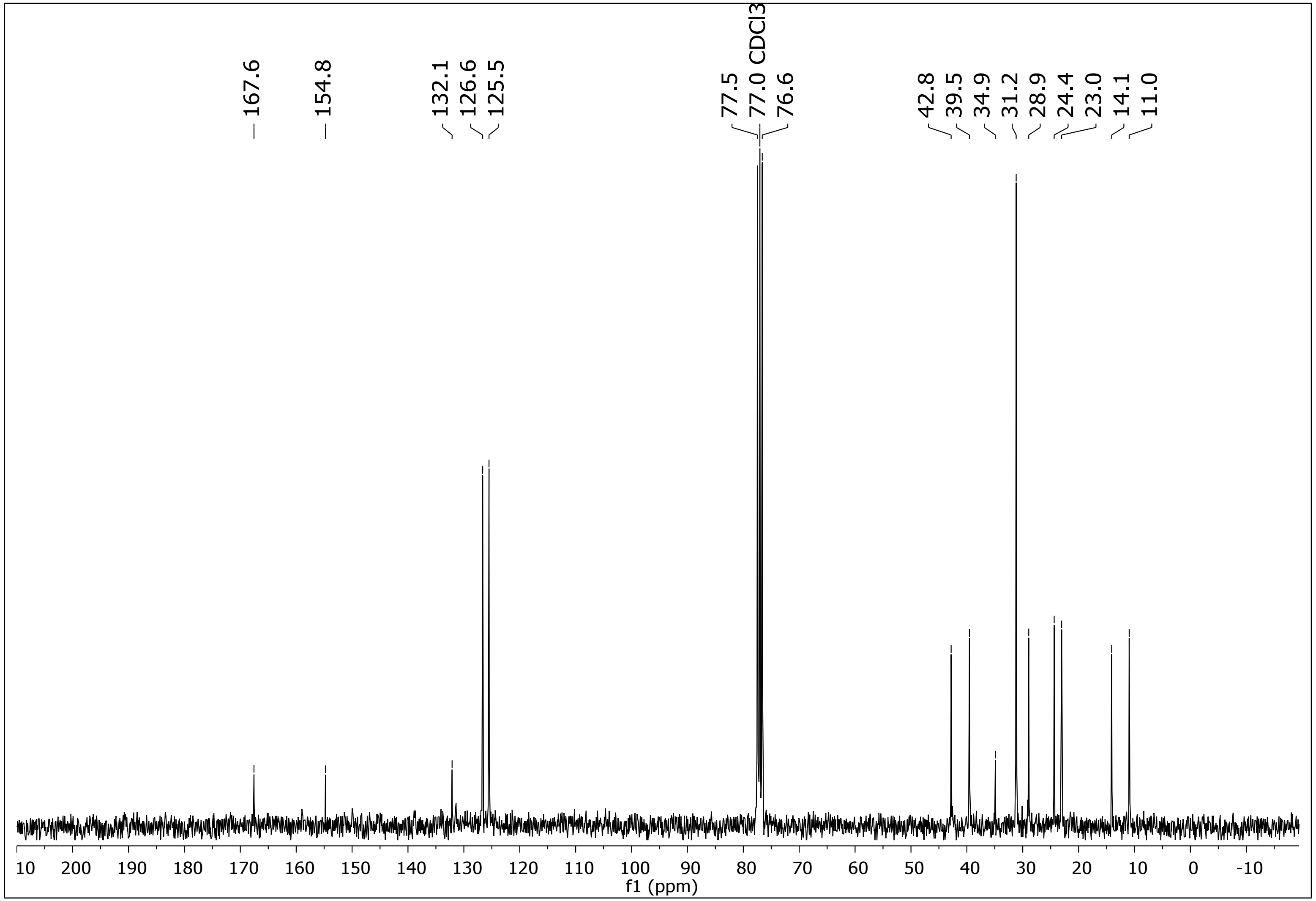

Figure S36. HRMS spectrum of compound **12**

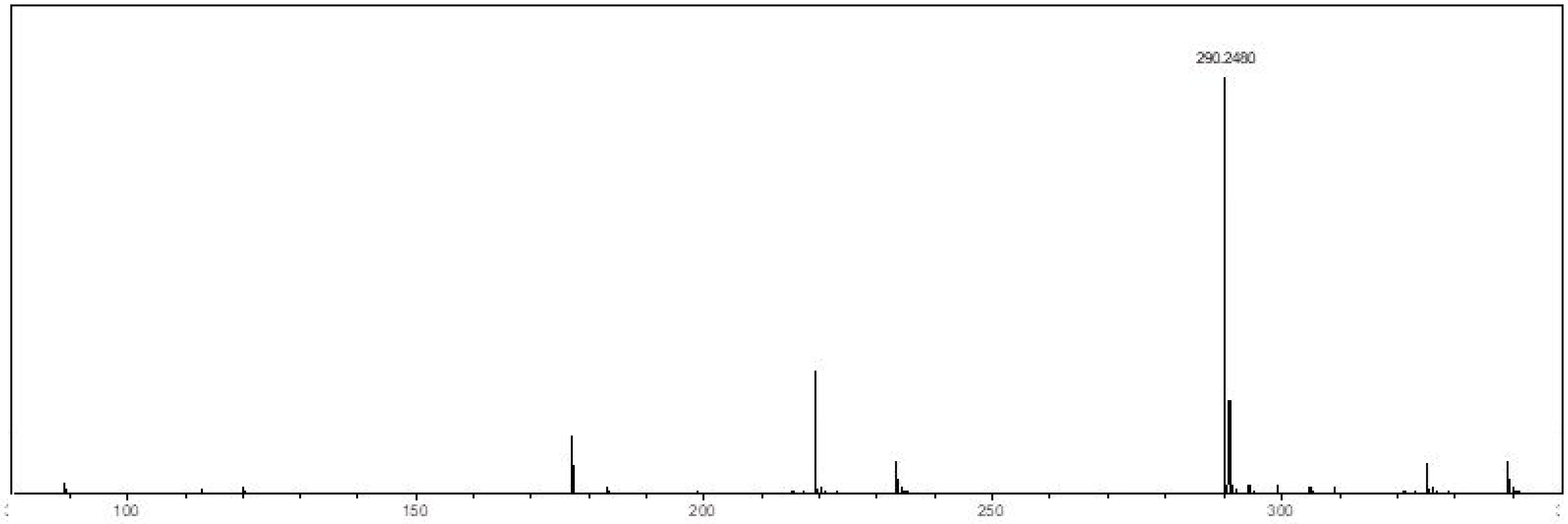

Supplement: Supplementary file 1 — ao4c05510_si_001.pdf [file ao4c05510_si_001.pdf]
